# The effectiveness of training in emergency obstetric care: a systematic literature review

**DOI:** 10.1093/heapol/czz028

**Published:** 2019-05-05

**Authors:** Charles A Ameh, Mselenge Mdegela, Sarah White, Nynke van den Broek

**Affiliations:** Centre for Maternal and Newborn Health, Department of International Public Health, Liverpool School of Tropical Medicine, Pembroke Place, Liverpool, UK

**Keywords:** Emergency obstetric care, newborn care, training, evaluation, effectiveness, health outcomes, systematic review

## Abstract

Providing quality emergency obstetric care (EmOC) reduces the risk of maternal and newborn mortality and morbidity. There is evidence that over 50% of maternal health programmes that result in improving access to EmOC and reduce maternal mortality have an EmOC training component. The objective was to review the evidence for the effectiveness of training in EmOC. Eleven databases and websites were searched for publications describing EmOC training evaluations between 1997 and 2017. Effectiveness was assessed at four levels: (1) participant reaction, (2) knowledge and skills, (3) change in behaviour and clinical practice and (4) availability of EmOC and health outcomes. Weighted means for change in knowledge and skills obtained, narrative synthesis of results for other levels. One hundred and one studies including before–after studies (*n* = 44) and randomized controlled trials (RCTs) (*n* = 15). Level 1 and/or 2 was assessed in 68 studies; Level 3 in 51, Level 4 in 21 studies. Only three studies assessed effectiveness at all four levels. Weighted mean scores pre-training, and change after training were 67.0% and 10.6% for knowledge (7750 participants) and 53.1% and 29.8% for skills (6054 participants; 13 studies). There is strong evidence for improved clinical practice (adherence to protocols, resuscitation technique, communication and team work) and improved neonatal outcomes (reduced trauma after shoulder dystocia, reduced number of babies with hypothermia and hypoxia). Evidence for a reduction in the number of cases of post-partum haemorrhage, case fatality rates, stillbirths and institutional maternal mortality is less strong. Short competency-based training in EmOC results in significant improvements in healthcare provider knowledge/skills and change in clinical practice. There is emerging evidence that this results in improved health outcomes.

**Key Messages**

- Short competency-based training in emergency obstetric care results in significant improvements in healthcare provider competence and change in clinical practice.
- There is emerging evidence that short competency-based training results in improved health outcomes.

## Introduction

Reducing maternal and neonatal mortality and morbidity globally remains a priority for the health and development agenda, in the Sustainable Development Goals (World Health Organization, 2015). Most maternal and newborn deaths and stillbirths occur during or immediately after labour and childbirth. With an increasing number of births now occurring at a healthcare facility even in low- and middle-income settings, current strategies focus on improving the quality of care during this critical period.

The minimum care package required during pregnancy and childbirth for the management of potentially life-threatening complications is referred to as emergency obstetric care (EmOC) (World Health Organization, 2009). The components (or signal functions) of this care package were agreed by the global partners in 1997 (World Health Organization, 2009). The EmOC care package addresses the main causes of maternal death, stillbirth and early neonatal death, including obstetric haemorrhage, (pre-)eclampsia, sepsis, complications of obstructed labour, complications of miscarriage or abortion and intrapartum asphyxia (Box 1). However, the EmOC signal functions include only one for newborns (resuscitation with bag and mask), other researchers have argued for a more comprehensive set of signal functions (Emergency Obstetric and Newborn signal functions) that includes care for small and sick newborns (Gabrysch *et al*., 2012; Moxon *et al*., 2015).

In-depth assessments of availability and coverage of EmOC have shown that in many cases the required infrastructure (including equipment and consumables) is available. However, staff may lack competency to provide all EmOC signal functions (Harvey *et al.*, 2007; Adegoke and van den Broek, 2009; Utz *et al.*, 2013). The combination of lack of knowledge and skills has been highlighted as a key reason why many beneficial evidence-based practices are still not in place (Tsu and Coffey, 2009).

Building the capacity of healthcare providers via ‘in-service’ or ‘on the job’ training has become a common approach. Such training is provided in almost all settings. Regular training is recommended and, in some cases, mandatory, to ensure continued accreditation of healthcare providers. In addition, many intervention programmes for maternal and newborn health in low- and middle-income countries (LMIC) include training of healthcare providers in EmOC as a significant component of their workplan and budgets (Nyamtema *et al.*, 2011).

Although training packages and teaching methodologies may vary there is a commonality of approach and a need for robust evaluation of the effectiveness of training (Penny and Murray, 2000; Black and Brocklehurst, 2003; van Lonkhuijzen *et al*., 2010). In their systematic review of models of training for labour ward personnel in acute obstetric emergencies, Black and Brocklehurst highlighted the need for research, an exact description of training programmes and a clear framework for monitoring and evaluation (Black and Brocklehurst, 2003). Evaluation of the effectiveness of training is important to improve training programmes and to provide information on how these can be developed and delivered to have the desired effect (Kirkpatrick, 1996; Kirkpatrick and Kirkpatrick, 2007).

We conducted a systematic review of studies that have evaluated the effectiveness of training in EmOC or components of EmOC. We developed a four-level framework to summarize data for the effectiveness of training with regard to: (1) participant reaction, (2) knowledge and skills, (3) change in behaviour and clinical practice and (4) availability of EmOC and health outcomes.

## Methods

### Search strategy

A protocol-driven systematic review was performed for the period between January 1, 1997 and December 31, 2017 using the recommended method for systematic reviews and reporting based on Preferred Reporting Items for Systematic Reviews and Meta-Analysis (PRISMA).

Both free-text and Medical Subject Headings (MeSH) terms were included in the search strategy including terms in three categories related to (1) emergency (essential, complication, life-saving), (2) participant, content, duration (obstetric, midwifery, neonatal, newborn infant, perinatal, post-partum, maternal, pregnancy, multidisciplinary) and (3) training or simulation (simulation, scenario, drill, virtual, education, meeting, workshop). We restricted our search to publications in English.

We searched PUBMED, EMBASE, COCHRANE library, SCOPUS, IMEMR (Eastern Mediterranean Index Medicus), ERIC, CINHAL (EBSCO), International trial register, Clinical trials.gov and the University of Liverpool library database (DISCOVER). ‘DISCOVER’ has both electronic and print collections, with access to theses, historical collections, 11 major databases (including EBSCO host, Medline, ProQuest, Scopus and web of science), access to publishers of major medical journals (and e-books) including JSTOR, Oxford, SAGE, Science Direct, Springer link, Taylor and Francis online and the Wiley online library.

The websites of key international organizations known to be involved with EmOC training (World Health Organization-WHO, United National Fund for Population Activities-UNFPA, United Nations Children Fund-UNICEF, John Hopkins Program for International Education in Gynecology and Obstetrics-JPHIEGO, Pathfinder, Population Council, Advanced Life Support Group, Childhealth Advocacy International, International Confederation of Midwives-ICM, International Federation of Obstetrics and Gynecology-FIGO, Royal College of Obstetrics and Gynaecology London-RCOG and African Ministry of Health websites) were searched for relevant reports and web-based publications. The reference lists of all relevant publications were reviewed for relevant articles not identified in the initial search. Finally, researchers and organizations were contacted by email for unpublished work that was relevant to the review.

### Inclusion or exclusion of studies

After the initial search, duplicates were excluded, and records identified were then screened by the researchers for eligibility, initially by title and then by the abstract. Reports which described the evaluation of in-service EmOC training programmes or courses and/or including any of the components of EmOC such as neonatal resuscitation were included. Publications were excluded if they were conference abstracts, study protocols, literature reviews of training methodology and equipment, or related to training for traditional birth attendants.

Two authors independently reviewed the full-text articles, when there was a disagreement, the paper was reviewed by and discussed with a third author.

### Grading of studies

The strength of the evidence was appraised using the Oxford Centre for Evidence-Based Medicine (OCEBM, 2011) levels of evidence.

### Analysis of findings

We used the Kirkpatrick’s framework for assessment of effectiveness of adult education in combination with the internationally agreed indicators for availability and quality of EmOC (Kirkpatrick and Kirkpatrick, 2007; World Health Organization, 2009; Ameh and van den Broek, 2015) to obtain data on the effectiveness of training at four levels: (Level 1) participants’ reactions to training, (Level 2) change in knowledge and/or skills, (Level 3) change in behaviour or clinical practice and (Level 4) change in the availability and quality of EmOC and in maternal and/or neonatal health outcomes.

Data were extracted from all included papers into a predesigned summary table with the following headings: author, year and country of publication, study objective, content of EmOC training, number and cadre of trainees, study design/evaluation approach, summary of results and quality of evidence (Supplementary Table 1: Summary table of all included studies).

A narrative synthesis approach was used due to the considerable heterogeneity of the included studies in terms of methodology, cadre of trainees, type of EmOC training and outcome measures obtained (Popay *et al.*, 2006). A formal meta-analysis was not possible as published data provided only means pre- and post-testing but did not include information on the variability of changes (with the exception of one study). However, for training that lasted between 1 and 5 days (inclusive) and, where scores for knowledge and skills were provided, weighted mean scores (aggregated across all areas assessed) for pre-training knowledge and skills and change after training for each were derived.

## Results

**Figure 1:**
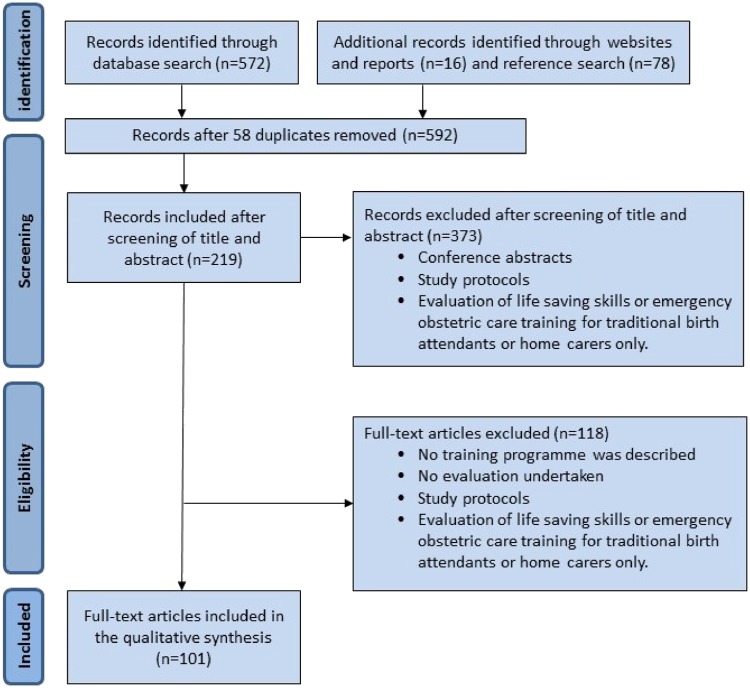
PRISMA flow diagram for systematic review of emergency obstetric care training packages.

The primary search produced 572 records. Additionally, 78 records were identified through websites and review of citations in published papers. After exclusion of duplicates and screening of titles and abstracts, 219 potentially eligible publications were identified. After full review, 118 papers or reports were excluded as these did not meet inclusion criteria. Finally, 101 papers or reports were included in this review; 13 systematic reviews with a mix of RCTs and cohort studies (OCEBM level 1a), 13 RCTs (OCEBM level 1b), 51 before–after studies (OCEBM level 2b), 13 longitudinal and retrospective studies (OCEBM level 3b) and three qualitative studies (Figure 1: PRISMA diagram).

**Figure 2:**
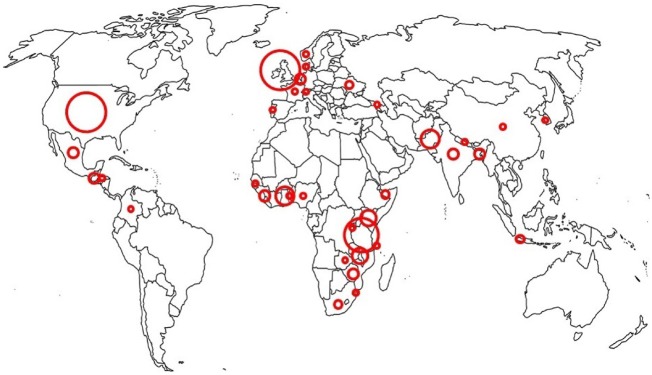
Geographical distribution of studies.

Excluding the systematic reviews identified, 50 of the papers included in this review were from LMICs and 38 from high income country (HIC). Before–after studies were the commonest and include 34 studies from LMIC and 10 from HICs. Only three RCTs were reported from LMICs in contrast to 12 from HICs (Figure 2: Bubble graph).

### Types of EmOC training

Duration of training ranged from 2 min covering one component of EmOC (newborn resuscitation) (Mazza *et al.*, 2017) to 24 weeks for the additional training of medical doctors to provide comprehensive EmOC (Gill and Ahmed, 2004). Five training packages were designed for delivery to one specific healthcare provider cadre only, while 16 training packages were designed for multidisciplinary teams, involving mostly medical doctors and nurses/midwives. Longer trainings were associated with a clinical component, including supportive supervision, feedback and retraining based on recognized need after initial training (Taylor, 1996; McDermott *et al.*, 2001; Gill and Ahmed, 2004; Osei *et al.*, 2005; Maslovitz *et al.*, 2007; Msemo *et al.*, 2013; Xu *et al.*, 2014; York *et al.*, 2014).

### Evaluation of the effectiveness of EmOC training

In total, 26 of the included studies assessed participants’ reaction (Level 1) and 42 assessed knowledge and skills (Level 2). In 51 (58%) studies, evaluation included the effect of training on clinical practice (Level 3). Almost a quarter of the studies (21) reported outcomes pertaining to health service provision or health outcomes (Level 4). Only 3 (<1%) studies assessed effectiveness at all four levels (Ameh *et al.*, 2012; Xu *et al.*, 2014; Marshall *et al.*, 2015).

In all settings, participant reaction was evaluated using questionnaires while for assessment of change in knowledge and skills, multiple choice questions, objectively structured clinical examinations or simulation-based tests were conducted.

Clinical practice was assessed using log books (Zaeem-ul-Haq *et al.*, 2009;Zafar *et al.*, 2009; Cole-Ceesay *et al.*, 2010), through interviews (Mavalankar *et al.*, 2009; Ameh *et al.*, 2012; Sakeah *et al.*, 2014), and focus group discussions (Ameh *et al.*, 2016), and via questionnaires sometimes administered as postal surveys (CAI, 2010; Zaeem-ul-Haq *et al.*, 2009), via telephone or via observation of practice (Taylor, 1996; CAI, 2007; Evans *et al.*, 2009; Freeth *et al.*, 2009; CAI, 2010; Crofts *et al*., 2013; Ersdal *et al.*, 2013; Makene *et al.*, 2014; Opiyo and English, 2015; Ortner *et al.*, 2014; York *et al.*, 2014; Dresang *et al.*, 2015; Walton *et al.*, 2016). Other methods and indicators used to assess change in healthcare providers’ clinical practice after training included retrospective analysis of clinical records Clark *et al.*, 2010), audit of clinical records or databases (Harris *et al.*, 1995; Dijkman *et al.*, 2010; Markova *et al.*, 2012) and review of the partograph (Taylor, 1996; Berglund *et al.*, 2010). Measures assessed include change in labour augmentation rate (Berglund *et al.*, 2010), change in episiotomy rate (Spitzer *et al.*, 2014; Dresang *et al.*, 2015), change in post-partum haemorrhage rate (Sørensen *et al.*, 2011; Spitzer *et al.*, 2014; Dresang *et al.*, 2015), number of perimortem caesarean sections per year (Dijkman *et al.*, 2010), caesarean section rate (Berglund *et al.*, 2010; Makuwani *et al.*, 2010; Sørensen *et al.*, 2011; Van de Ven *et al.*, 2016), vacuum delivery rate (Sørensen *et al.*, 2011; Dresang *et al.*, 2015), change in obstetric referral pattern (Ronsmans *et al.*, 2001; Nielsen *et al.*, 2007; Makuwani *et al.*, 2010; Nyamtema *et al.*, 2016; Rahman *et al.*, 2017), diagnosis and management of EmOC complications (Varghese *et al.*, 2016), quality of face mask ventilation (Mazza *et al.*, 2017), degree of implementation of protocols (Deering *et al.*, 2004; Xu *et al.*, 2014; Burstein *et al.*, 2016; Kim *et al.*, 2016; Van de Ven *et al.*, 2016) and documentation of achievement of strategic goals to change practice post-training (Walker *et al.*, 2012, 2014). At the health system level, the availability and/or change in EmOC signal functions were assessed in three studies (Evans *et al.*, 2009; Ameh and van den Broek, 2015; Dresang *et al.*, 2015).

Health outcomes assessed include Neonatal Mortality Rate (Carlo *et al.*, 2010; Sørensen *et al.*, 2011; Msemo *et al.*, 2013; Walker *et al.*, 2016), perinatal mortality rate (Spitzer *et al.*, 2014), case fatality rate (CFR) (Nyamtema *et al.*, 2011; Spitzer *et al.*, 2014; Ni Bhuinneain and McCarthy, 2015), institutional maternal mortality ratio (iMMR) (Dresang *et al.*, 2015; Ni Bhuinneain and McCarthy, 2015), fresh stillbirth rate (Nyamtema *et al.*, 2011; Msemo *et al.*, 2013), stillbirth rate (Draycott *et al.*, 2006; Carlo *et al.*, 2010; Nyamtema *et al.*, 2011; Sørensen *et al.*, 2011), maternal mortality due to post-partum haemorrhage (Burstein *et al.*, 2016; Van de Ven *et al.*, 2016), incidence of birth asphyxia (Draycott and Crofts, 2006; MacKenzie *et al.*, 2007; Carlo *et al.*, 2010; Xu *et al.*, 2014; Varghese *et al.*, 2016), occurrence of neonatal complications after shoulder dystocia (Inglis *et al.*, 2011; MacKenzie *et al.*, 2007; Grobman *et al.*, 2014; Van de Ven *et al.*, 2016) and retrospective assessment of Apgar scores, stillbirth rates and hypoxia ischaemic encephalopathy (Ameh *et al.*, 2012).

Results obtained for each level of effectiveness are provided below.

### Level 1: Participants’ reaction to training

About 30% (*n* = 26) of all studies evaluated participants’ reaction (18% or 28% of all evaluations in LMIC and 8% or 33% of all evaluations in HICs). This was often done if a new training programme was being introduced and participants’ reaction was used to make adaptations to the training package (Ameh *et al.*, 2012; Nelissen *et al.*, 2014). Specific issues addressed included requests from participants for longer periods of training and translation of training materials into the local language. The largest multi-country study in sub-Saharan Africa included 600 participants and reported that healthcare providers gave high scores for acceptability and enjoyment of training and considered the content useful in their setting to improve quality of care (Ameh *et al.*, 2012). Participant reaction assessed in other settings was similarly positive including in India (Evans *et al.*, 2009; Mavalankar *et al.*, 2009; Varghese *et al.*, 2016), Indonesia (McDermott *et al.*, 2001), China (Xu *et al.*, 2014), Bangladesh (Johanson *et al.*, 2002a), Armenia (Johanson *et al.*, 2002a), Tanzania (Nelissen *et al.*, 2014), Mexico (Walker *et al.*, 2012, 2014, 2016), Guatemala (Walker *et al.*, 2015; Walton *et al.*, 2016), Pakistan (Zaeem-ul-Haq *et al.*, 2009), the USA (Marshall *et al.*, 2015), the UK (Freeth *et al.*, 2009; Howie *et al.*, 2011), Switzerland (Monod *et al.*, 2014) and Denmark (Markova *et al.*, 2012).

### Level 2: Change in knowledge and skills

Forty-two (48%) studies included evaluation of knowledge and skills of participants, usually comparing scores before the training with those obtained immediately after. Most studies reported improved knowledge and skills immediately after the training. Very few studies used a set pass mark to judge improvement (Ronsmans *et al.*, 2001; Nielsen *et al.*, 2007; Ersdal *et al.*, 2013; Conroy *et al.*, 2014). Evans *et al.* (2009) reported no improvement in scores for those trainees who had a high pre-test score. One large before–after study (5939 healthcare workers in seven countries) reported on the change in scores relative to pre-training scores (mean improvement ratio-IR) and examined factors associated with pre-training scores and IR (Conroy *et al.*, 2014). In this study, 99.7% of participants had improved scores, with a median score increase of 10% (interquartile range 5–10%). A teaching job, previous training in EmOC and a higher percentage of time spent providing maternity care were associated with higher pre-training scores. Five studies used a control group, and all reported significant improvement in knowledge and skills in the intervention groups (5–20% difference) immediately after training (McDermott *et al.*, 2001; Crofts *et al.*, 2007b; Frank *et al.*, 2009; Crofts *et al.*, 2013; Xu *et al.*, 2014).

**Figure 3:**
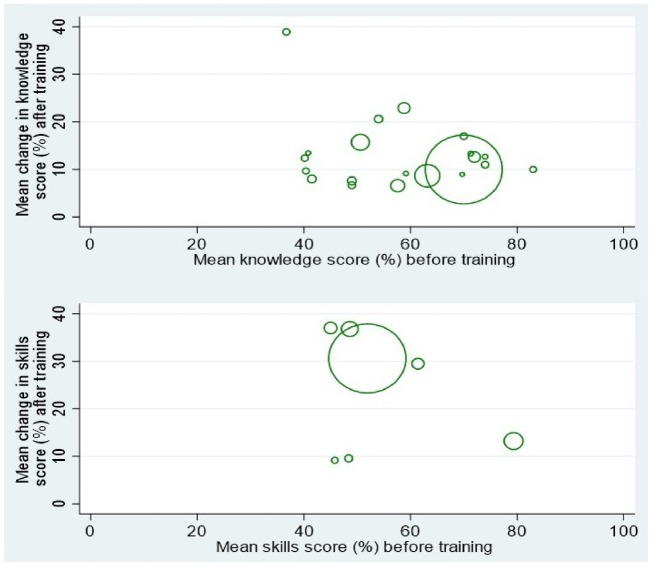
Change in knowledge and skills calculated by pre-training score.

A total of 13 studies provided data used to provide an aggregate analysis of change in knowledge and skills. Studies were included if EmOC training was 1–5 days (inclusive) and assessment scores were provided (Table 1: Studies providing data for aggregated analysis). Change in knowledge and skills was calculated by pre-training score (Figure 3: Scatterplot). The weighted mean pre-training score was 67% for knowledge with a mean change of 10.6% and 53.1% for skills with a mean increase of 29.8% were obtained. Confidence intervals (CIs) could not be calculated as 12 of the 13 studies did not provide information on standard deviation for the mean change in scores.

**Table 1:** Studies included in aggregated analysis for change in knowledge and skills after training in emergency obstetric care

Authors, Year and Country of study	Study objective	Training duration and content	Study design/ Evaluation approach	Sub-population where applicable	Knowledge/ Practical skills	Pre- Test	Post- Test	Change	Number of trainees in the evaluation
Ameh *et al*. (2012) Somaliland	To evaluate in-service training in ‘Life Saving Skills—Emergency Obstetric and New-born Care’	3-day training designed to cover the five major causes of maternal deaths	A before–after study was conducted using quantitative and qualitative methods		Knowledge	57.6	64.2	6.6	183
					Skills	45	82	37	140
Ameh *et al*. (2016) Ghana, Nigeria, Sierra Leone, Malawi, Kenya, Tanzania, Zimbabwe, Bangladesh and Pakistan	Evaluation of knowledge and skills of maternity care providers after EmOC training	3–5 days	Before–after study		Knowledge	70	80	10	5757
		Simulation-based training in emergency obstetric and early newborn complications	Multiple choice questions and objective structured clinical examination was used						
					Skills	51.9	82.5	30.6	5161
Evans *et al*. (2014) Malawi, Zanzibar and Tanzania	To validate the Helping Mothers Survive: Bleeding After Birth training module	1-day	Before–after study	Malawi	Knowledge	83	93	10	42
		Facility-based training on management of post-partum haemorrhage		India	Knowledge	70	87	17	47
				Zanzibar	Knowledge	74	85	11	50
Grady *et al*. (2011) Kenya, Malawi, Somaliland, Swaziland, Zimbabwe, Tanzania and Sierra Leone	To evaluate the effect of LSS-EOC and NC training	3-days	Before–after study		Knowledge	63.2	71.8	8.6	600
		Five major causes of maternal deaths early newborn care	Assessment of knowledge and skills training						
					Skills	48.6	92.6	44	600
Homaifar *et al*. (2013) Rwanda	To determine improvement in knowledge and skills among medical students after completion of Advanced Life Support in Obstetrics (ALSO) training	2 days	Before–after study		Knowledge	54	74.6	20.6	65
		Five major causes of maternal deaths	Assessment of knowledge and skills training						
Moran *et al*. (2015) South Africa	To describe the scale up of Emergency Obstetric and Newborn Care training in one province in South Africa	ESMOE: multi-disciplinary, simulation-based ‘skills and drills’ using training of trainers approach	Before–after study		Knowledge	36.7	75.6	38.9	45
			Assessment of knowledge and skills training						
Tang *et al*. (2016) Malawi	To evaluate whether a hospital-based mentoring programme could significantly increase Emergency Obstetric and Newborn Care (EmONC) knowledge and skills	1 day	Before–after study Written and practical tests		Knowledge	58.8	81.7	22.9	134
		Training and mentoring on EmONC using skills laboratory							
Varghese *et al*. (2016) India	Limited effectiveness of a ‘skills and drills’ intervention to improve emergency obstetric and newborn care	2 days	Quasi experimental study design	Obstetric complications	Knowledge	49	56.6	7.6	73
					Skills	45.8	55	9.2	35
		Emergency drills and skills refresher training	Before and after knowledge and skills assessment						
				Newborn resuscitation	Knowledge	49	55.7	6.7	50
					Skills	48.4	58	9.2	50
Walker *et al*. (2012) Mexico	Evaluation of the PRONTO course	3 days	Before–after study		Knowledge	41.5	49.5	8	68
		Obstetric haemorrhage, eclampsia and neonatal resuscitation							
Walker *et al*. (2014) Mexico	Simulation-based obstetric and neonatal care. PRONTO course	3 days	Before–after study		Knowledge	50.6	66.3	15.7	305
		PRONTO, simulation-based obstetric and neonatal emergency team training							
					Skills	79.4	92.6	13.2	305
Maslovitz *et al*. (2007) Israel	Simulation-based training for labour ward and delivery teams involved in obstetric emergencies	1 day	Quasi experimental study design		Knowledge	69.7	78.7	9	18
		Simulation-based training on managing common obstetric emergencies							
			Videotape and use of checklists to assess competence						
Crofts *et al*. (2007b) United Kingdom	To explore the effect of obstetric emergency training on knowledge and to assess if acquisition of knowledge is influenced by the training setting or teamwork training	1 or 2 days	Randomized controlled trial	Junior doctors	Knowledge	40.8	54.3	13.5	21
		Life-saving skills in emergency obstetric care							
			Intervention group data	Senior doctors	Knowledge	59.1	68.3	9.2	22
				Junior midwives	Knowledge	40.4	50.1	9.7	44
				Senior midwives	Knowledge	40.2	52.5	12.3	46
Crofts *et al*. (2008) United Kingdom	To explore the effect of training on patient actor perception of care during simulated obstetric emergencies	1 or 2 days	Randomized controlled trial	Post-partum haemorrhage	Knowledge	71.3	84.6	13.3	24
		Life-saving skills on eclampsia and PPH management	Intervention group data						
				Eclampsia	Knowledge	74	86.7	12.7	24
				Shoulder dystocia	Knowledge	72	84.6	12.6	132
Weighted means aggregated across all studies					Knowledge	67.0	77.6	10.6	7750
					Skills	53.1	82.9	29.8	6054

#### Retention of knowledge and skills

Eight studies assessed knowledge and skills in the medium to longer term following training in EmOC (Ronsmans *et al.*, 2001; Basnet *et al.*, 2004; CAI, 2010; Yang *et al.*, 2012; Crofts *et al.*, 2013; Homaifar *et al.*, 2013; Tang *et al.*, 2016). The systematic review by Yang *et al.* (2012) assessed resuscitation skills only and concluded that knowledge and skills decline after 6–12 months with skills declining faster than knowledge. Child-health Advocacy International in a cluster randomized study (36 participants) reported that improvement in knowledge observed immediately after training in EmOC was not sustained at 6 months post-training (CAI, 2010). In contrast, a RCT in the UK noted sustained knowledge [related to management of shoulder dystocia, eclampsia and post-partum haemorrhage (PPH)] at both 6 and 12 months post-training (Crofts *et al.*, 2013). Monod *et al.* (2014) reported a significant reduction in knowledge attained by 3 months after training with scores ranging from 96.9% to 36.3%. Similarly, there is evidence that scores for communication skills decrease over time; Ronsmans *et al.* (2001) reported a reduction of 78% at 3 months to 64% at 16 months post-training, respectively. Only one study (before–after study in one institution) from a LMIC evaluated knowledge and skills retention after training which included all the EmOC signal functions, at 3 and 6 months (Tang *et al.*, 2016). The immediate post-training improvement in knowledge was not retained by 6 months (*n* = 111, difference 3.1%, *P* < 0.001) but was retained for skills (*n* = 111, difference 1.7% *P* < 0.054) (Cooper *et al.*, 2011). Tang *et al.* (2016) reported that lack of opportunities to put into practice what was learned and associated with a significant reduction in knowledge and skills at 6 months post-training.

### Level 3: Change in clinical practice

Almost 60% (*n* = 51) of all included studies assessed the effectiveness of training with regard to change in clinical practice. These studies measured adherence to protocols and evidence-based practice, resuscitation of mother and baby, teamwork and communication and performance of peri-mortem caesarean section.

#### Adherence to protocols and evidence-based practice

Berglund *et al.* (2010) in a before–after study, reported a change in clinical practice 3 months after training in effective perinatal care with a 4-fold increase in the use of the partograph. A before–after study in Tanzania, that observed practice and measured post-partum blood loss 7 weeks before and 7 weeks after ALSO training, reported that training of healthcare providers significantly improved the active management of the third stage of labour and decreased the number of episiotomies performed (Sørensen *et al.*, 2011). Spitzer *et al*. (2014) also reported a reduction in episiotomies performed after a 5-day training in EmOC in Kenya.

Opiyo and English (2015) concluded that there was moderate evidence that in-service neonatal emergency care training improves treatment of seriously ill babies by health professionals in the short term.

Two retrospective cohort studies (Inglis *et al.*, 2011; Van de Ven *et al.*, 2016), one standard-based review (Burstein *et al.*, 2016), post-training simulation (Maslovitz *et al.*, 2007) and one before–after study (Grobman *et al.*, 2014), showed improved competency, adherence to management protocols (Maslovitz *et al.*, 2007; Inglis *et al.*, 2011; Burstein *et al.*, 2016) and improved documentation of procedures (Grobman *et al.*, 2014; Burstein *et al.*, 2016; Van de Ven *et al.*, 2016). Varghese *et al.* (2016), in a quasi-experimental study, implemented and evaluated training in four intervention and four control sites. Knowledge and skills improved immediately after training but diagnosis and management of EmOC did not improve. Barriers to improved diagnosis and management identified were staff attrition and irregular supply of EmOC drugs and supplies (Varghese *et al.*, 2016).

#### Resuscitation of the newborn or mother

Two studies evaluating training in resuscitation of the baby at time of birth reported improved ventilation technique and adherence to resuscitation protocols (Xu *et al.*, 2014; Mazza *et al.*, 2017). A before–after study by Mazza *et al.* (2017), assessed the effectiveness of face-mask ventilation following 2-min training, feedback after assessment and further 2-min training, measuring ventilatory parameters using a computerized system. There was a significant decrease in mask-leak after the training (t1: 19.5%, SD: 32.8%, t2: 39.2% SD: 37.7%), reducing further after additional 3-min verbal recall plus real-time feedback.

Opiyo *et al.* (2008) in an RCT (*n* = 83) evaluating newborn resuscitation training reported an improvement in clinical practice and a reduction in harmful or inappropriate practices. However, an evaluation of the Helping Babies Breathe programme using a before–after study design (*n* = 53) reported that improved knowledge and skills after training in newborn resuscitation training did not result in a change in clinical practice (Ersdal *et al.* 2013). Conroy *et al.* (2014) made similar observations in a cross-sectional study on the competence of trainees in newborn resuscitation (follow-up time not specified).

Two studies reported on the number of resuscitation attempts following training. In a cohort study evaluating 234 healthcare workers trained in Essential Surgical Skills with Emphasis on Emergency Maternal and Child Health in Pakistan using log books to document resuscitation attempts (Zafar *et al.*, 2009). Zafar *et al.* (2009) reported a response rate (proportion of trainees who completed the logbooks) of 53% and a total of 1123 resuscitation attempts by medical doctors (76%) and nurses (24%) for adults (including pregnant women) as well as babies and children, with a survival rate of 89%. Similarly, in Gambia after training 217 healthcare providers, there were 293 documented resuscitation attempts reported after the training, but the response rate was not reported (Cole-Ceesay *et al.*, 2010). However, in both studies, there was no control group and no baseline number of resuscitation attempts before training (Zafar *et al.*, 2009; Cole-Ceesay *et al.*, 2010).

A retrospective cohort study following the introduction of the Managing Obstetric Emergencies and Trauma (MOET) course in the Netherlands reported a statistically significant increase in peri-mortem caesarean sections conducted after the introduction of the course (from 0.36 to 1.6 per annum; *P* = 0.01) (Dijkman *et al.*, 2010).

#### Communication and teamwork

Walker *et al.* (2014), in a before–after study, involving 12 matched pairs of hospitals (450 healthcare professions trained at the intervention sites) reported that there was significant improvement in knowledge, self-efficacy and teamwork scores in the intervention sites.

A systematic review on the effect of multidisciplinary teamwork training (including four RCTs and four cohort studies). Merién *et al.* (2010) showed improved practical skills, knowledge, communication and team performance in acute obstetric situations following simulation-based multidisciplinary team training in EmOC. No additional benefit was reported from additional training focusing on teamwork or teambuilding alone (Birch *et al.*, 2007; Cooper *et al.*, 2011). One RCT evaluated the impact of team work specific training (with no obstetric skills training) on the occurrence of adverse outcomes. While no significant difference was noted in the mean adverse outcome index between intervention and control arms, the ‘decision to incision time’ for caesarean section was reduced from 33.3 to 21.2 min after training (*P* = 0.03), suggesting improved communication as a result of team training (Walker *et al.*, 2012). In contrast a RCT study involving 24 medical doctors and midwives (in four teams two in each arm) evaluated the effect of a separate training in teamwork (with or without training in EmOC) using directed commands during communication as the outcome measure. Teams that received additional team work training used more directed commands suggesting that specific team training may add value to multidisciplinary training in EmOC (Siassakos *et al.*, 2009).

### Level 4: Availability and quality of EmOC and health outcomes

Of the included studies, four specifically evaluated change in the availability of EmOC or component signal function, whereas 17 studies reported on perinatal or maternal health outcomes.

#### Availability of EmOC

Ameh *et al.* (2012) in a before–after study (*n* = 222) reported an increase in availability and performance of EmOC signal functions at healthcare facilities designated to provide Basic or Comprehensive EmOC (an increase of 43% and 56%, respectively). Similarly, Evans *et al.* (2009) in a study from India reported that the number of healthcare facilities providing all EmOC signal functions increased from 2 to 10 among 15 facilities included in the study. An increase in the number of midwives providing selected signal functions—indicative of task shifting—was reported by Ameh *et al.* (2012) (manual vacuum aspiration and assisted vaginal delivery) and Clark *et al.* (2010) (post-abortion care, PAC).

A retrospective evaluation of the impact of PAC training for 434 midwives and 53 medical doctors (trained separately) in Ghana showed that after training, availability of PAC was increased with both doctors and midwives providing this service (previously mainly doctors). The proportion of trained midwives providing PAC was lower than for doctors (20% vs 80%). Factors associated with midwives’ offering PAC services were; working in healthcare facilities where the national reproductive health standards and policy was available, and working in private healthcare facilities (Fransen *et al.*, 2012).

Two before–after studies assessed the effectiveness of programmes which included training in EmOC and reported an increase in the number of women who accessed a healthcare facility for birth (skilled birth attendance) (78% and 100% increase) and a decrease (66%) in obstetric referrals indicative of improved availability of EmOC (Nyamtema *et al.*, 2016; Rahman *et al.*, 2017).

#### Quality of EmOC

There is evidence to suggest training in EmOC improves client satisfaction. An RCT to explore the effect of training on clients’ perceptions of care during simulated obstetric emergencies reported significantly higher scores for safety (*P* = 0.048) and a trend towards better communication (*P* = 0.77) for teams trained onsite using an actor compared with teams trained at a simulation centre using a computerized patient mannequin (Crofts *et al.*, 2008). Training in EmOC via both self-paced learning, and using the traditional on-site approach, resulted in improved interaction between service providers and women attending for antenatal care and improved client satisfaction regarding waiting times (McDermott *et al.*, 2001).

In the evaluation of the PRONTO training programme in Mexico, asked participants to set specific goals related to improvement in the quality of maternal and perinatal care services. In this study, Walker *et al.* (2014) reported 60% achievement of these goals, 3 months after the training; however, there was no association between goal achievement and change in knowledge scores.

## Maternal and perinatal health outcomes

A before–after study (7 weeks before and 7 weeks after) conducted in a regional hospital in Tanzania, reported a decrease in the number of women who had PPH from 32.9% to 18.2% [risk ratio (RR) 0.55 (95% CI 0.44–0.69)], and severe PPH from 9.2 to 4.3% [RR 0.47 (95% CI 0.29–0.77)] (Sørensen *et al.*, 2011). Spitzer *et al.* (2014) reported similar results from Kenya with the occurrence of PPH reduced from 3.3% (*n* = 1740) to 2.3% (*n* = 1802) *P* < 0.001). CFR was the stated primary outcome measure in the study from Tanzania but no data were provided. Nyamtema *et al.* (2011) in a review of maternal health programmes that included training in EmOC, reported a 40% and 50% reduction in CFR and Maternal Mortality Ratio, respectively. (Bhuinneain and McCarthy, 2015) in a systematic review that included eight studies, concluded that there was moderate evidence that training in EmOC reduced the iMMR and CFR. Dresang *et al.* (2015) showed reduced maternal mortality after ALSO training in Colombia (before–after study, *n* = 35), Guatemala (case–control study, *n* = 1609). However, Spitzer *et al.* (2014) reported no change in iMMR.

In the UK, a large retrospective cohort study showed a statistically significant reduction in the number of babies born with low Apgar scores at 5 min (≤6, *P* < 0.001) and in incidence of neonatal hypoxic-ischaemic encephalopathy (*P* = 0.032) with no change in stillbirth rates following 1-day training of maternity staff (Draycott and Crofts, 2006). Berglund *et al.* (2010) in Ukraine, reported a reduced number of hypothermic babies and admissions into the neonatal unit but no effect on early neonatal mortality following three-day training in Effective Perinatal Care. A number of non-RCTs reported a reduction in the number of hypothermic babies and admission into neonatal intensive unit (Spitzer *et al.*, 2014), neonatal mortality rate (Carlo *et al.*, 2010; Sørensen *et al.*, 2011; Msemo *et al.*, 2013; Walker *et al.*, 2016) and fresh stillbirth rate (Msemo *et al.*, 2013). Conversely, two studies reported no decrease in stillbirth rate (Carlo *et al.*, 2010; Sørensen *et al.*, 2011). One cluster RCT reported reduced incidence of asphyxia related deaths in the delivery room following training in neonatal resuscitation (Xu *et al.*, 2014).

A before–after study by Grobman *et al.* (2014) and a retrospective cohort study by Inglis et al. (2011) both evaluating training for shoulder dystocia showed a reduction in brachial plexus palsy at delivery (10.1–2.6%, *P* < 0.3), and reduced incidence of brachial plexus injury (30–10.7% *P* < 0.1).

## Discussion

Training in EmOC is provided in many countries to ensure healthcare providers working in maternity care are able to recognize women who have complications during pregnancy or at the time of birth, and to prevent and manage these complications effectively such that maternal and perinatal morbidity and mortality is reduced. These training packages are designed for several healthcare provider cadres including nurse-midwives, general medical doctors, anaesthetists and specialist obstetrician-gynaecologists. Often, training in EmOC is multidisciplinary with the specific aim to also improve communication and teamwork. There are a variety of training programmes available which are of different length, but the majority are short (1–5 days) and are provided as ‘skills and drills’ or simulation-based training.

### Main findings

This systematic review summarizes the studies that have evaluated the effectiveness of training in one or more components of EmOC. There is very good evidence that healthcare providers enjoy this type of training and find it relevant to their day-to-day work and clinical settings (Level 1). There is also strong evidence to show that competency is increased with statistically significant improvements in knowledge and skills in the majority of healthcare providers who attend for training (Level 2). There is good evidence of improved clinical practice including with regard to adherence to protocols for care and evidence-based practice (Level 3). However, overall, there is much fewer data to support that this is translated into improved health outcomes (Level 4). There are high-quality studies from high-income settings that demonstrate that training in specific aspects of care (e.g. the timely recognition and correct management of shoulder dystocia) results in decreased neonatal morbidity. Similarly, there are some studies mostly from low- or middle- income setting that demonstrate improved recognition and management of common complications, e.g. improved prevention of PPH and reduction in the number of women with PPH and severe PPH. However, there are very few high-quality studies that are able to demonstrate that training in EmOC results in a reduction in stillbirths, CFRs, neonatal or maternal mortality. This is likely, at least in part, because such studies are difficult to design and conduct.

### Strengths and limitations

It is important to evaluate the effectiveness of training at different levels. For this systematic review, a well-defined four-level evaluation framework was used incorporating the methodology for evaluation of adult-learning proposed by Kirkpatrick and Kirkpatrick (2007), and the framework and indicators to assess availability and quality of EmOC defined by the UN (World Health Organization et al., 2009). Thus, data obtained from studies were extracted to include effectiveness (or not) with regard to healthcare provider acceptability, knowledge, skills, change in practice and EmOC availability as well as maternal and perinatal health outcomes. In general, stronger evaluation designs (involving control groups or RCTs) have been used in HIC compared with LMIC. Only 3 of 88 included studies, provided data for all four levels of effectiveness that need to be assessed (Ameh *et al.*, 2012; Xu *et al.*, 2014; Marshall *et al.*, 2015). Studies were heterogeneous with regard to design and measures of effectiveness obtained and, EmOC training packages had varying content. This limited the analyses that could be performed with a narrative synthesis rather than meta-analysis conducted for the majority of data obtained. However, for the first time, aggregated weighted means are provided for pre-training level of knowledge and skills and change after training. These indicate that both are significantly improved but generally skills are more improved than knowledge—a result that can be expected given the focus of training packages in EmOC on acquisition of skills and clinical practice, and the mode of delivery which is largely simulation- or ‘skills and drills’-based.

### Implications for practice

There are a variety of EmOC training programmes available which have been implemented with success and demonstrably strengthen healthcare provider competence and team working. Several of the EmOC training packages were developed in HIC and have been adapted for delivery in LMIC. Multidisciplinary training is effective in improving healthcare provider knowledge, skills, communication and team working (Birch *et al.*, 2007; Siassakos *et al.*, 2009; Merién *et al.*, 2010, Cooper *et al.*, 2011). The organization, content and method of delivery of in-service training programmes is critical for their effectiveness. On-site training improves communication within obstetric teams, but, is not more effective than off-site training. However, there are reports that off-site training is more acceptable to healthcare providers than on-site training (Crofts *et al.*, 2006; Crofts *et al.*, 2008; Guise and Segel, 2008; Daniels *et al.*, 2010). A combination of lectures (smaller proportion of content) and simulation-based or ‘skills and drills’ participatory training appears to result in greater improvement in team performance (improved knowledge, skills and confidence) than lectures or didactic teaching alone (Black and Brocklehurst, 2003; Crofts *et al.*, 2006; Crofts *et al.*, 2007a,b; Crofts *et al.*, 2008; Guise and Segel, 2008; Daniels *et al.*, 2010; Cooper *et al.*, 2011).

There is limited data to suggest what the optimum length of a training package (including all EmOC components) should be. Only two before–after studies (both from LMIC) were identified which compared EmOC training programmes of different durations. Longer training programmes were associated with greater improvement in skills compared with shorter programmes (McDermott *et al.*, 2001; Osei *et al.*, 2005). Peer support and on-the-job supervision contributed to improved skills in the longer versions of the training packages evaluated (McDermott *et al.*, 2001; Osei *et al.*, 2005; Msemo *et al.*, 2013).

Data on the retention of knowledge and skills over time are useful to determine how frequently healthcare providers should be ‘re-trained’ to maintain competency in EmOC. In this systematic review, eight studies reported on retention. The majority of studies that have evaluated knowledge and skills retention after EmOC training have been in high resourced settings (Harris *et al.*, 1995; Black and Brocklehurst, 2003; Crofts *et al.*, 2006; Crofts *et al.*, 2007a; Yang *et al.*, 2012). Knowledge and skills can be retained for up to 1-year post-training and healthcare providers report being confident to provide EmOC for up to 1-year post-training. Skills decline at a faster pace than knowledge. Factors that affect retention of skills and knowledge include previous relevant clinical experience, repeat training and opportunities to practice new skills. It was not surprising that there were no studies assessing knowledge and skills retention >12 months because there will be several confounding factors to account for.

While there is a need to adapt training packages to specific contexts, they should be designed based on evidence. These training packages should be routinely evaluated at all levels using robust study designs to improve the potential for scale up.

A multi-country study including six LMIC specifically designed to measure retention after training in EmOC observed that mean scores for knowledge and skills at each of four quarterly subsequent assessments remained between those immediately post-training and those at 3 months. They concluded that healthcare providers retain knowledge and skills for up to 12 months (Ameh *et al.*, 2018).

Some obstetric complications are not common and depending on caseload, health care workers may not see such complications regularly. To retain the ability to correctly manage such complications, they should have the opportunity to have ‘booster’ training at regular intervals (Ameh *et al.*, 2018; Sutton *et al.*, 2011).

### Implications for research

Study designs recommended by the Cochrane Effective Practice and Organization of Care Groups for inclusion in educational systematic reviews are RCTs, non-RCTs, controlled before–after studies and interrupted time series (ITS) (Cochrane Effective Practice and Organization of Care Group, 2013). Of the 101 studies in this review, 51 were ITS or before–after studies, and 3 were qualitative studies. Ten of the RCTs were conducted in HIC and only three in LMIC settings.

While overall, there are a good number of studies which have evaluated the effectiveness of training programmes in the last 15 years, data on the effectiveness (or not), in particular, pertaining to maternal and perinatal health outcomes, is still very limited. Such studies would require robust study designs and would require the inclusion of control sites or populations, or, use randomized allocation of training (vs no training). Additionally, adverse health outcomes including maternal mortality are less frequent and would require large sample sizes. Even with skilled healthcare providers in place, a reduction in mortality cannot be expected to occur unless there is a functional health system including adequate resources, organization and financing in place (Roemer, 1993). Therefore, it could be assumed that change in health outcomes can be expected to be associated with, but would be difficult to attribute to, EmOC training per se in any programme evaluation, and that this is not a realistic outcome to expect or that can be measured.

Measurements of change in knowledge and skills should, wherever possible, correct for the individual participants’ pre-training scores (and thus improvement potential) and this may be a more objective method to assess improvements than a simple before–after comparison of scores. A wide range of methods and measures was used to assess change in clinical practice. Agreement on measures of clinical practice such as compliance with agreed standards for specific defined complications or morbidities, and more consistent measurement of these would be beneficial and allow for better comparison across studies and settings.

## Conclusions

There is evidence that short competency-based training in EmOC results in increased knowledge and skills of healthcare providers and in improved clinical practice. A supportive working environment and referral pathway, as well as opportunities to practice skills, are likely to promote longer-term retention of knowledge and skills obtained after training. There is evidence that training in EmOC results in more components of this care package being available including in LMIC settings and that maternal and perinatal health outcomes are improved as a result.

Emergency obstetric and newborn care training packages are a means to ensure that women and newborns have competent health care providers for routine care and to manage complications. This is in line with the WHO standards for improving the quality of care in health facilities. This is an essential pathway to achieving the ambitious maternal and newborn health SDG targets.

## Supplementary Material

czz028_Supplementary_Data
