# Supplementary material for: The effectiveness of training in emergency obstetric care: a systematic literature review
Source: Health Policy Plan. 2019 May 5;34(4):257–70. doi: 10.1093/heapol/czz028 (PMC6661541; doi:10.1093/heapol/czz028)
Supplement: czz028_Supplementary_Data [file czz028_supplementary_data.docx]

**Supplementary Supporting Table 1: Summary of findings - Studies included in systematic review providing information on evaluation of effectiveness of training in Emergency Obstetric Care (EmOC)**

| **Authors, Year and Country of study** | | **Publication reference** | **Study objective** | **Training content** | **Number of trainees included in evaluation** | **Study design/Evaluation approach** | **Summary of results** | **Quality of evidence** |
| --- | --- | --- | --- | --- | --- | --- | --- | --- |
| **Systematic Reviews** | | | | | | | | |
| **Ameh and van den Broek, 2015** | | Best Practice & Research Clinical Obstetrics and Gynaecology 29(2015):1077-91 | To identify key elements of EmONC training and evaluation, and describe the Making it Happen Program | Training for healthcare providers in EmONC and evaluation methods | N/A | **Review**  18 peer reviewed articles representing 10 EmONC training courses/packages and 7 peer reviewed articles describing evaluation approaches  **Level 1** | Evidence supports training approaches utilizing simulated ‘skills and drills’, should be of short duration, as close to the working environment as possible and based upon adult learning methodology. | 2a |
| **Bergh et al, 2015** | | Best Practice & Research Clinical Obstetrics and Gynaecology 29(8):1028-43 | To map the landscape regarding training in EmONC skills and give an overview of the different training programs, packages and approaches | Training for healthcare providers in EmONC | N/A | **Review**  35 peer reviewed articles representing 23 studies/trials assessed by Kirkpatrick level (K1-4) and training methods  K2 (4 studies)  K3 (3 studies)  K4 (8 studies)  Methods (8 studies)  **Levels 1, 2, 3, 4** | Training programs had all been developed in high-income countries and adapted for use in low- and middle-income countries. Most studies showed positive results. Evidence favors onsite, multi-disciplinary, team-based training using realistic, low-technology equipment for simulation. | 2a |
| **Bhuinneain and McCarthy, 2015** | | BJOG: An International Journal of Obstetrics and Gynaecology 122(2):174-82 | To investigate the presence and rigor of evidence for effective capacity building for Essential Obstetric and Newborn Care (EONC) to reduce maternal mortality in rural, sub-Saharan Africa | Training for healthcare providers in emergency obstetric care |  | **Systematic Review**  22 studies included of which   - 4 studies assessed impact of training on perinatal outcomes - 4 studies examined the effect of capacity-building programs in EmONC on maternal mortality and demonstrated a reduction in institutional mortality and a halving of case-fatality rates   **Level 4** | There is moderate evidence to support the training of healthcare workers of different cadres in the provision of emergency obstetric and newborn care services to reduce institutional maternal mortality and case-fatality rates in rural sub-Saharan Africa. | 2a |
| **Black and Brocklehurst, 2003** | | BJOG: An International Journal of Obstetrics and Gynaecology 110(9):837-41 | Description of in-service emergency obstetric training packages and evaluation of training (developed country setting only) | MOET and ALSO training packages | MOET-UK: 30 ALSO-USA: 1012 ALSO-USA: 275 ALSO-USA: 55  Midwives, obstetric trainees, consultants in obstetrics and anesthesia, family physicians | **Systematic Review**  4 studies included; before-after comparison of reaction, comfort and confidence responding to obstetric emergencies  **Level 1** | Improved comfort and confidence was sustained at 10 and 12 months in 2 studies. There was no study comparing one type of training with another. | 2a |
| **Calvert et al, 2013** | | Australian and New Zealand Journal of Obstetrics and Gynaecology 53(6):509-16 | To identify the evidence for the clinical impact of simulation training in obstetrics emergency training | Obstetric emergency training | Skilled healthcare workers | **Review**  92 articles included of which  59 were commentary or review articles  20 had non-clinical evidence of the impact of training  13 had Clinical evidence of the impact of training  Kirkpatrick’s level of training evaluation was used.  **Levels 1, 2, 3, 4** | Most studies were evaluated at KL1-3, 4 studies were evaluated at KL4 only and 1 study was evaluated at KL1-3. There was evidence of positive impact of obstetric training in eclampsia, shoulder dystocia, post-partum hemorrhage, maternal collapse, cord prolapse and teamwork training. However, this is mostly at participant’s level rather than clinical outcome level. | 2a |
| **Cooper et al, 2011** | | Women and Birth 25(2):64-78 | Examine the evidence for simulation-based learning in midwifery education | Evidence from obstetrics, neonatology, technical and non-technical skills (teamwork) training was included where it informed the development of midwifery curricula | Not specified | **Systematic Review**  24 papers included, all were quantitative studies: 6 interrupted time series, 9 cohort studies, 7 RCTs, 2 systematic reviews  **Level 3** | The benefits of simulation compared to didactic-based learning are apparent. This approach also aids the development of non-technical skills, confidence and competence. | 1a/2a |
| **Meaney et al, 2010** | | Resuscitation 81(11):1462-72 | Systematic review to evaluate whether the inclusion of any specific resuscitation training educational strategy in developing countries improved outcomes | Resuscitation training | Not specified | **Systematic Review**  44 papers included 15 of good quality, 17 of fair quality and 12 of poor quality; only 1 RCT, 4 non-randomized crossover studies, 21 retrospective controlled studies, 10 non-controlled studies and 2 descriptive studies.  **Levels 1, 2, 3, 4** | 38/44 papers supported the use of resuscitation training programs in developing countries. Increased patient survival after resuscitation with absolute risk reduction ranging 0%-34%. There was no consistent testing method for educational outcomes across studies and few studies examined educational outcomes and health outcomes. 15 studies assessed self-efficacy and 8 studies assessed student satisfaction. 15 studies assessed patient survival, 14 of which were either newborn or trauma resuscitation or only 1 was adult resuscitation. | 2a |
| **Merién et al, 2010** | | Obstetrics and Gynecology 115(5):1021-31 | Effectiveness of multi-disciplinary teamwork training in a simulation setting for the reduction of medical adverse outcomes in obstetric emergency situations | Teamwork training programs with simulation models for labor ward staff in acute obstetric emergencies | Not specified | **Systematic Review**  86 studies included; 8 studies evaluated the effectiveness of team training (4 RCTs and 4 cohort studies)  **Levels 1, 2, 3** | Teamwork training in a simulation setting resulted in improvement of knowledge, practical skills, communication, and team performance in acute obstetric situations. | 1a/2a |
| **Nyamtema et al, 2011** | | BMC Pregnancy & Childbirth 11(30):94-102 | Impact of maternal health interventions in resource limited countries | Multiple intervention programs which included: training in EmONC, deployment of healthcare providers, refurbishment of healthcare facilities, infrastructure, improved supply of drugs, equipment and consumables for obstetric care | Not specified | **Systematic Review**  58 papers included: 35 of 54 multiple intervention programs reviewed included EmOC training, placement and motivation of healthcare providers; effect of EmOC training alone not assessed  **Level 4** | Programs integrating multiple interventions were likely to have significant positive impacts on maternal outcomes. Births and caesarean section rates in EmOC facilities increased by 71-75%, case fatality rates and maternal mortality ratios reduced significantly by 40% and 50%, respectively. | 2a |
| **Opiyo and English, 2015** | | The Cochrane Database of Systematic Reviews 2015(5):CD007071 | To assess the effects of in-service emergency care training on health professionals’ treatment of seriously ill newborns and children in low-income countries | In-service emergency care training in neonatal and child care |  | **Systematic Review - first update on previous review**  2 studies included (RCTs)  Two randomized trials met the eligibility criteria  **Levels 3, 4** | There was improvement in performance of adequate initial resuscitation steps (risk ratio 2.45, 95% CI 1.75, 3.42, p<0.001) and reduction in the frequency of inappropriate and potentially harmful practices (mean difference 0.40, 95% CI 0.13, 0.66, p=0.004).  There is moderate evidence that in-service neonatal emergency care courses probably improve health professionals’ treatment of seriously ill babies in the short term. More studies are, however, needed to assess long-term effect of in-service emergency neonatal care training. | 1a |
| **Rabøl et al, 2010** | | Quality and Safety in Health Care 19(6):e27 | Outcome of classroom-based multi-professional team training for hospital staff | Team training | Not specified | **Systematic Review**  18 studies included: 15 studies were not controlled. 17 studies had a moderate or high risk of bias  **Levels 1, 2, 3, 4** | Participant reaction, learning and behavior change as well as effect on clinical processes was positive in all studies, but for some only partially. Results at patient level were limited. More than half of the studies ended evaluation within 6 months. | 2a |
| **van Lonkhuijzen et al, 2010** | | BJOG: An International Journal of Obstetrics and Gynaecology 117(7):777-87 | To assess the effectiveness of training programs aimed at improving emergency obstetric care in low-resource environments | Training programs aimed at improving emergency obstetric care in low-resource environments | Not specified | **Systematic Review**  38 papers included; large variation the study designs, training content and method of evaluation  Frequently poor study design and non-validated measurement tools used  **Levels 1, 2, 3, 4** | Positive reaction, increased knowledge and skills and improved behavior after training were reported while health outcomes were infrequently reported. | 2a |
| **Yang et al, 2012** | | Resuscitation 83(9):1055-60 | To evaluate the retention of adult advanced life support (ALS) knowledge and skills following completion of an ALS course in healthcare providers. | Adult life support training | Nurses, physicians, anesthesiologist, newly graduated doctors (n=10-325) | **Systematic Review:**  Data from included articles analyzed using a structured approach and organized outcomes by evaluation method and knowledge and skills retention  336 articles retrieved, 11 papers were included  3 RCTs, 5 repeated measure quasi-experimental study, 3 descriptive observational studies and 1 descriptive correlational study; 10 study designs were of fair quality and 1 was poor  **Level 2** | Most studies used multiple choice questionnaires to evaluate knowledge retention and cardiac arrest simulation or other skills tests to evaluate skills retention. All studies reported variable rates of knowledge or skills deterioration over time, from 6 weeks to 2 years after training. Two studies noted retention of knowledge at 18 months and up to 2 years, and one reported skills retention at 3 months. Clinical experience, either prior to or after the courses, has a positive impact on retention of knowledge and skills.  Conclusion: ALS knowledge and skills decay by 6 months to 1 year after training and that skills decay faster than knowledge. | 2a |
| **Low- and Middle-Income Countries** | | | | | | | | |
| **Ameh et al, 2012**  **Somaliland** | International Journal of Gynecology and Obstetrics 117(3):283-7 | | To evaluate in-service training in “Life Saving Skills – Emergency Obstetric and New-  born Care” | 3-day training designed to cover the 5 major causes of  Maternal deaths: hemorrhage, sepsis, eclampsia, obstructed labor and complications of abortion; newborn resuscitation and early newborn care, all 9-signal functions of EmOC | 222 healthcare providers | A **Before-after study** was conducted using quantitative and qualitative methods to evaluate trainee reaction and change in knowledge, skills and behavior, in addition to functionality of healthcare facilities, during and immediately after training, and at 3- and 6-months post-training.  **Levels 1, 2, 3** | The healthcare providers reported improved conﬁdence in providing EmOC. Basic and comprehensive EmOC healthcare facilities provided 100% of expected signal functions compared with 43% and 56%, respectively, at baseline with  trained midwives performing skills usually performed by medical doctors after the training. | 2c |
| **Ameh et al, 2016**  **Ghana, Nigeria, Sierra Leone, Malawi, Kenya, Tanzania, Zimbabwe, Bangladesh and Pakistan** | PLoS ONE 11(12):e0167270 | | Evaluation of knowledge and skills of maternity care providers after EmOC training | Emergency obstetric and early newborn complications, identification and treatment. Predominantly simulation-based training methods | 5,939 healthcare workers | **Before-after study design.**  Multiple choice questions and objective structured clinical examination was used. The outcome measures were change in knowledge and skills scores, and improvement ration. Linear regression was performed to identify variables associated with pre-training scores and improvement ration  **Level 2** | 99.7% of all healthcare providers improved their overall score and IR, median (IQR) increase of 10% (5-15%). There was significant improvement in knowledge and skills for each cadre and by country. The mean IR for medical doctors was 56%, midlevel staff 50% and nurse-midwives, 38% for nursing aides. A teaching job, previous EmOC training and higher percentage of time spent providing maternity care were associated with higher pre-training scores. The largest iR was for recognition and management of obstetric haemorrhage, while the smallest IR was for the use of the partograph. | 2c |
| **Basnet et al, 2004**  **Nepal** | International Journal of Gynecology and Obstetrics 86(1):98-108 | | To evaluate the effectiveness of post-abortion care (PAC) training | Emergency treatment of complications, Manual Vacuum Aspiration for first trimester abortions, post procedure family planning, counselling. Medical doctors training also included use of D&C for gestation >12 weeks | 24 nurses and 17 medical doctors | **Cohort study**  Pre- and post-training knowledge test, availability of components of PAC, number of PAC cases managed, over a 1-year period  **Levels 2, 3** | Follow-up of 6 nurse-providers in 2 district hospitals; all 6 scored 85% or above for knowledge. Family planning and counselling services not previously available under medical doctor-led services were available post-training in the 2 hospitals. The number of PAC cases increased by 37%. The proportion of referred cases to the next level of care did not change significantly (10% vs. 11%). This was attributed to staff redeployment after training. | 2b |
| **Berglund et al, 2010**  **Ukraine** | Acta Obstetricia et Gynecologica Scandinavica 89(2):230-7 | | To describe the process of change and assess compliance and effect on maternal and infant outcomes of WHO training package on Effective Perinatal Care (EPC) | Didactic, interactive 2-week training package including clinical practice and a team approach to maternity care | Obstetricians, neonatologists, midwives, pediatric nurses, pediatricians and anesthesiologists | **Intervention study** comparing outcomes before and after training, 3 maternities, for 2.5 years  **Levels 3, 4** | EPC procedures were successfully implemented and adherence to the protocols was excellent. For most variables, the change occurred during the first 3 months and was well sustained. Use of partograph increased between 60 and 400%; induction and augmentation of labor decreased to <1% and 5%, caesarean section rate decreased signiﬁcantly in 2 maternities, proportion of hypothermic infants decreased from 60-85%% to 1% in all three maternities, admission to Neonatal Intensive Care Unit decreased in 2 maternities. There was no effect on early neonatal mortality. | 2b |
| **Carlo et al, 2010**  **Zambia** | Paediatrics 126(5):1064-71 | | To test the hypothesis that two training programs would reduce incrementally 7-day neonatal mortality rates for low-risk institutional deliveries | ENC training: Routine neonatal care, resuscitation, thermoregulation, breast-feeding, "kangaroo" (skin-to-skin) care, care of the small baby, and common illnesses  Neonatal resuscitation program: Basic resuscitation knowledge, skills, bag and mask ventilation and chest compressions | Midwives in 18 clinics | **Before-after study**  Data collected during 3 months after implementation of each training course  **Level 4** | 71,689 neonates were enrolled over 3 months. 7-day neonatal mortality rate decreased from 11.5% to 6.8% deaths per 1,000 live births after training (relative risk: 0.95% CI: 0.48-0.77; p<0.001), due to the decrease in deaths from birth asphyxia and infection. There was no decrease in stillbirth rates. There was a further decrease in neonatal mortality rates after further Neonatal Resuscitation Training. ENC training for midwives reduced 7-day neonatal mortality rates in low-risk first referral level, urban community clinics. | 2c |
| **Child Health Advocacy International, 2007**  **Pakistan** | **Report**  Published by Child health Advocacy International, Islamabad, Pakistan:  Evaluation of ESS-EMCH training in Pakistan | | Evaluation of ESS-EMCH training in Pakistan, to determine its impact on knowledge, attitudes and practice | Essential Surgical Skills -Emergency Maternal and Child Health (ESS-EMCH) training package | 234 healthcare providers: 194 medical doctors and 40 nurse-midwives | **Before-after study**  Comparison of knowledge and skills, postal survey post training  **Levels 1, 3** | Improvement in knowledge and skills: pre-course knowledge test - doctors: 63.52+/-13.32, nurses: 54.89+/-18.31. Post-course knowledge test- doctors: 71.46+/-8.87, nurses; 65.47+/-7.68. (p=0.00)  Qualitative survey: 54% response rate. Increased confidence, frequent use of skills, bag and mask for neonatal resuscitation and hand washing. | 2b |
| **Child Health Advocacy International, 2010**  **Pakistan** | **Report**  Published by Child health Advocacy International, Islamabad, Pakistan  Evaluation of Essential Surgical Skills with emphasis on Emergency Maternal and Child Health Program by assessing doctors' practices in Pakistan | | To evaluate the impact of Essential Surgical Skills-Emergency Maternal and Child Health (ESS-EMCH) training in Pakistan | Essential Surgical Skills -Emergency Maternal and Child Health (ESS-EMCH) training package | Observational study  7 medical doctors 36 medical doctors in public sector hospitals of 3 districts were involved in the evaluation  18 each in the intervention and control groups  284 patients, 124 in each group | Phase 1: **Cluster RCT**  Phase 2: **Observational** study: 6-months post-training, knowledge and skills assessment  **Levels 1, 2, 3** | Knowledge scores immediately after training were significantly increased (p=0.03) with non-significant change in knowledge 6 months after training (p=0.20).  There was a statistically significant difference in the number of patients treated using the structured approach between the intervention; 63.7% (79) and control 37.1% (46) arms (OR 2.98, 95%CI 1.78-4.99, p=0.0001). Cluster analysis revealed similar findings for doctors: intervention-62.9% (50.4-75.3) vs. control-36.3% (26.3-46.4) (p=0.001)  Observational study results: There was significant retention of skills 6 months after the training. | 1b |
| **Clark et al, 2010**  **Ghana** | Journal of Midwifery & Women's Health 55(2):153-61 | | The impact of training in Post Abortion care (PAC) on service provision | Post Abortion Care | 53 medical doctors and 434 nurse-midwives | 5-year **retrospective analysis** of secondary data; assessment of change in behavior and service provision  **Level 3** | Overall, 28% of providers were offering PAC services (medical doctors 80%, nurse midwives 20%). In bivariate analysis, provision of PAC services was associated with training. Working in a facility with the National Reproductive Health Standards and Policy available or a private healthcare facility was associated with midwives offering PAC services. | 2c |
| **Cole-Ceesay et al, 2010**  **Gambia** | Reproductive Health 7(1):21 | | To describe and evaluate interventions to improve emergency care | Training in the recognition and management of emergencies based on WHO tool kit, ALSG UK and Essential Surgical Skills with Emphasis on Emergency Maternal and Child Health (ESS-EMCH training package) | 217 doctors, nurses and nurse-midwives | **Cohort study**: log books used to document resuscitation attempts over 30-month period following training  **Level 3** | A total of 293 resuscitation attempts documented in logbooks, response rate not reported. | 2c |
| **Conroy et al, 2014**  **Sierra Leone** | Resuscitation 85(2):e31-e32  Letter | | Evaluation of baseline retention of previous training in neonatal resuscitation training of healthcare workers in Bo District, Sierra Leone | Not described, nor interval between training and evaluation | Not clear if all those assessed were originally trained but 42 healthcare workers were observed:  5 Midwives,  18 Nurses,  4 Community Health Workers,  10 Maternal and Child Health Aides  3 Community Health Assistants and 2 traditional birth attendants | **Cross-sectional study**  Experienced neonatal resuscitation trainers observed healthcare workers and graded their performance as excellent, identified need for successfully commenced resuscitation and inadequate resuscitation.  It was not clear if observation of real cases was combined with simulation using a model  **Levels 1, 2** | The score, proportion of staff with that score and the 95% confidence interval were 36 or 86% CI (72-93) of healthcare workers were graded as inadequate resuscitation, 6 or 14% (7-28) identified the need for and commenced resuscitation and none of the healthcare workers were graded as excellent. | 4 |
| **Crofts et al, 2015**  **Zimbabwe** | Bulletin of World Health Organization 93(5):347-51 | | Evaluation of a hospital-based EmONC course, PROMPT in Mpilo Tertiary Hospital Zimbabwe | A hospital train-the-trainers course, followed by 1-day courses for 299 hospital staff in Practical Obstetric Multi-Professional Training (PROMPT) | 12 local trainers  299 hospital staff (Midwives, midwifery tutors, nurses, laboratory technicians, pharmacists, junior doctors, obstetricians, pediatricians, anesthetists and family doctors) including 138/153 (90%) of staff who worked within the maternity | **Before-after study**  Primary outcome measures  Numbers trained  % change in appropriate action to abnormal observations  Institutional maternal mortality  **Levels 3, 4** | After the score charts were introduced, appropriate action in response to abnormal observations (e.g. starting anti-hypertensives or antibiotics) had increased from 1/24 (4%) to 11/15 (73%) leading to a 34% reduction in maternal mortality at Mpilo Central Hospital from 67 maternal deaths per 9,078 live births (0.74%) in 2011 compared with 48 maternal deaths per 9,884 live births (0.49%) in 2014. | 2b |
| **Dresang et al, 2015**  **Colombia,**  **Guatemala,**  **Honduras and**  **Tanzania** | International Journal of Gynecology and Obstetrics 131(2):209-15 | | To examine the effects of the Advanced Life Support in Obstetrics (ALSO) program on maternal outcomes in four low-income countries | ALSO: 2-day EmONC training course with lectures and simulations | Colombia: 35 ICU intensivists  Guatemala: 1609 faculty, students and obstetrics and gynecology residents  Honduras: 54 participants (obstetricians, residents, students, nurse midwives, nurses)  Tanzania: 40 nurse-midwives, doctors and assistant medical officers | Colombia: **Cohort study**. Pre- and post-intervention maternal outcomes  **Level 4**  Guatemala: 1 intervention hospital vs 3 similar control hospitals, annual maternal outcome data  **Level 4**  Honduras: **Cohort study**, single center. Perceived self-efficacy, maternal outcome data, following training and at 6- and 12-months  **Levels 2, 3, 4**  Tanzania: **Prospective study**, single center, pre- and post-training active management of the third stage of labor (AMTSL) observation and postpartum hemorrhage (PPH) measurement  **Level 4** | Colombia: Training increased recorded maternal morbidity and near misses but reduced maternal mortality.  Guatemala: Training reduced in overall maternal mortality, and mortality from PPH.  Honduras: Training reduced episiotomy rates, increased AMTSL, vacuum-assisted delivery and reported self-efficacy.  Tanzania: Training reduced PPH and severe PPH. | 2b |
| **Ersdal et al, 2013**  **Tanzania** | Resuscitation 84(10):1422-7 | | To determine the effect on practical skills and **management** strategies among providers using simulations seven months after Helping Babies Breathe (HBB) training, and to describe neonatal **management** in the delivery room during the corresponding time period before/after a 1-day HBB training in a rural Tanzanian hospital | 1-day HBB (newborn resuscitation) training was conducted by Tanzanian master instructors in April 2010  There was no retraining or efforts to facilitate skill transfer and sustainability of performance after training was put in place | Cohort total: 53  Pre HBB: 39  Post HBB: 27 | **Before-after study**  39 healthcare providers performed two simulation scenarios; "routine care" and "neonatal resuscitation" were performed by before (September 2009) and 27 providers after (November 2010) the HBB training  Two independent raters scored the videotaped scenarios  Overall "pass/fail" performance and different skills were assessed. During the study time period (September 2009-November 2010) no HBB re-trainings were conducted  Observational data on neonatal management before (n=2,745) and 7 months after (n=3,116) the HBB training was collected in the delivery room by observing all births at the hospital during the same time period.  **Levels 2, 3** | The proportion of providers who "passed" the simulated "routine care" and "neonatal resuscitation" scenarios increased after HBB training, from 41 to 74% (p=0.016) and from 18 to 74% (p≤0.0001) respectively. However, the number of babies being suctioned and/or ventilated at birth did not change, and the use of stimulation in the delivery room decreased after HBB training. Improvements in skills observed during simulation testing were not transferred clinical practice | 2c |
| **Evans et al, 2009**  **India** | International Journal of Gynecology and Obstetrics 107(3):277-82 | | To evaluate the impact of 16-week  Comprehensive EmOC (CEmOC) training program for medical doctors | CEmOC training | 17 general medical officers | **Cohort study:** **Before-after study**  Primary outcome: Availability of EmOC signal functions  **Level 3** | More trainees performed each of the basic EmOC skills after the training than before. After training, 10 of 15 facilities to which trainees returned could provide all signal functions for basic EmOC whereas only 2 could do so before. For comprehensive EmOC, 2 facilities with obstetricians were providing all functions before and 2 were doing so after, even though the specialists had left those facilities and services were being provided by CEmOC trainee. | 2c |
| **Evans et al, 2014**  **Malawi, Zanzibar and Tanzania** | International Journal of Gynecology and Obstetrics 126(3):286-90 | | To validate the Helping Mothers Survive: Bleeding After Birth training module | 1-day healthcare facility-based training  Prevention, detection and management of post-partum training | 155 skilled and semi-skilled healthcare workers | **Before-after study**  **Levels 1, 2** | The proportion of providers with passing knowledge scores increased significantly from pre- to post-training among all cadres except for those already high at baseline. On three post-training skills tests the overall proportion of individuals with a passing score ranged from 83% to 89%. | 2c |
| **Frank et al, 2009**  **Republic of South Africa** | South African Journal of Obstetrics and Gynaecology 15(3):94-9 | | To assess the effect of the Essential Steps in Managing Obstetric Emergencies (ESMOE) training program on improving knowledge and skills of interns | 3- day training in management of obstetric emergencies | 68 medical interns undergoing rotation in obstetrics and gynecology from 8 hospitals | **Before-after study**  comparison of knowledge and skills using multiple choice questions and Objective Structured Clinical Examination methods of interns who had received in training in addition to rotation (intervention group) compared to interns who had done rotation only (control group)  **Level 2** | Knowledge scores (mean and (range)) of trained interns group were significantly higher than their pre-test scores and those of the control group (79 (58 - 93), 75 (48 - 91) and 77 (57 - 86) respectively). Similarly, the OSCE scores for skills of the intervention group were significantly higher than their pre-test scores and those of the control group (30.75 (25 - 38), 19.75 (8.5 - 27.5) and 24.5 (14.4 - 31) respectively. | 2c |
| **Grady et al, 2011**  **Kenya, Malawi, Somaliland, Swaziland, Zimbabwe, Tanzania and Sierra Leone** | Journal of Obstetrics and Gynaecology 31(1):18-23 | | To evaluate the effect of LSS-EOC & NC training | 3-day training designed to cover the 5 major causes of  maternal death (hemorrhage, sepsis, eclampsia, obstructed labor and complications of abortion) newborn resuscitation and early newborn care, all nine signal functions of EmOC. | 600 healthcare providers (nurse-midwives, medical doctors, clinical officers and specialists) | **Cohort study:** **Before-after study** training assessment of knowledge and skills  **Levels 1, 2** | Knowledge (n=600) about the diagnosis and management of complications of pregnancy and childbirth as well as newborn care significantly increased (p<0.001). There was measurable improvement in skills n=238 (p<0.001), and participants expressed a high level of satisfaction with the training. | 2c |
| **Homaifar et al, 2013**  **Rwanda** | International Journal of Gynecology and Obstetrics 120(2):195-9 | | To determine improvement in knowledge among MD6 students after completion of Advanced Life Support in Obstetrics (ALSO) training; to determine retention of knowledge from completion of the ALSO course to the final assessment; and to determine individual, structural, and environmental factors that might predict competence among students with regard to provision of quality obstetric care | ALSO course (obstetric emergencies) | 65 final-year medical students | **Quantitative assessments** to evaluate emergency obstetrics knowledge and practical skills were administered before, immediately after, and 3–9 months following the training course  **Level 2** | In total, 52 (80.0%) students demonstrated knowledge improvement after training. 57 (87.7%) students improved or maintained their scores from the post-training written test to the final assessment, and 32 (49.2%) retained practical skills. Twenty-one (32.3%) of the class demonstrated competency in both written and practical skills. According to multi-variable logistic regression analysis, female gender was associated with overall competency (p=0.01), and use of the internet for academic purposes more than 3-5 times per week tended toward competency (p=0.11) | 2c |
| **Johanson et al, 2002**  **Bangladesh** | BMC Medical Education 2:7 | | To evaluate the effectiveness of the Managing Obstetric Emergencies and Trauma (MOET) course in Bangladesh | 3-day training in MOET | 9 obstetrician-gynecologists | **Before-after study** comparing knowledge and management of an obstetric emergency scenario  **Levels 1, 2** | All participants showed an improvement in knowledge scores: the range of scores was narrower after- (143-175) compared to before training (69-146). After training none of the candidates had unsatisfactory scores for the scenario management. The overall ratings in anonymous evaluation of training were good. | 2c |
| **Johanson, Menon et al, 2002**  **Armenia** | Journal of Obstetrics and Gynecology Research 28(4):217-23 | | To evaluate the effectiveness of the Managing Obstetric Emergencies and Trauma (MOET) course in Armenia | 3-day training in MOET | 8 obstetrician-gynecologists | **Before-after study** Comparison of knowledge and management of an obstetric emergency scenario  **Levels 1, 2** | All participants showed an improvement in knowledge scores. The range was narrower after (167-188) compared to before training (85-129.5). In the scenario assessment, none of the participants had an unsatisfactory score. The majority of the comments were complimentary and positive. All candidates enjoyed the course, 7/8 felt strongly that they learnt new procedures and 5/8 felt strongly confident to practice and teach obstetric emergencies after training. | 2c |
| **Makene et al, 2014**  **Tanzania** | BMC Pregnancy & Childbirth 14:381 | | To evaluate the effects of a large-scale maternal-newborn quality improvement intervention in Tanzania that assessed the quality of provision of essential newborn care and newborn resuscitation | USAID-funded program with key programmatic approaches, including training of healthcare workers in BEmONC  and routine delivery care, supportive supervision, provision of equipment and quality improvement support to more than 1,593 providers and supervisors  from 251 facilities nationwide | 243 healthcare workers (nurses, midwives,  clinical officers and assistant medical officers) in 52 healthcare facilities assessed | **Before-after study**  Cross-sectional healthcare facility surveys were conducted pre-intervention (2010) and post-intervention (2012)  Observation of essential newborn care provided by healthcare providers immediately following the birth  Observation of actual management of newborns with trouble breathing  Assessments of healthcare worker knowledge  Facility audits to assess facility readiness for essential newborn care  **Levels 2, 3, 4** | Index scores for quality of observed essential newborn care showed significant overall improvement following the quality of care intervention, from 39% to 73% (p<0.0001).  Healthcare worker knowledge using a case study significantly improved as well, from 23% to 41% (p<0.0001) but skills in resuscitation using a newborn mannequin were persistently low.  Availability of essential newborn care supplies, which was high at baseline in the regional hospitals, improved at the lower-level healthcare facilities. | 2b |
| **Makuwani et al, 2010**  **Tanzania** | East African Journal of Public Health 7(2):109-13 | | To evaluate the impact of Emergency Obstetric Care (EmOC) training and advocacy on the availability and utilization of CEmOC | Management of prolonged and obstructed labour, obstetric haemorrhage, pre-eclampsia and eclampsia, puerperal sepsis and severe anaemia | 15 medical assistants, medical doctors and nurse-midwives | **Before-after study**  **Level 3** | The number of facility deliveries increased from 306 to 636 per month, institutional caesarean section rate 17.4% and the number of referrals out reduced by 80%. | 2c |
| **Mavalankar et al, 2009**  **India** | International Journal of Gynecology and Obstetrics 107(3):283-8 | | To evaluate the impact of the 18-week long Life Saving Anaesthetic Skills (LSAS) for Emergency Obstetric Care (EmOC) trained medical officers | Resuscitation in obstetric emergencies and provision of anaesthesia for obstetric procedures | 51 medical doctors | 14 **Qualitative** interviews using purposive sampling  **Level 1** | The training well received but trainees wanted more practice. Being posted with a specialist anesthesiologist and with a co-operative EmOC provider increases the likelihood that participants would provide anaesthesia. The working environment in healthcare facilities is insufficient to tackle emergency obstetric procedures. |  |
| **Mazza et al, 2017**  **Togo** | PLoS ONE 12(10):e0186731 | | To evaluate a short training to improve the quality of face-mask ventilation (FMV) in a low resource setting | New born face-mask ventilation 2-minute training using a mannequin | Twenty-six subjects participated in the study | **Before and after study**  T1=before  T2=after 2 minute training  T3= after further 2-minute training with feedback  **Primary outcome**: % of breaths with relevant mask leak (>25%)  **Secondary outcomes: %** of breaths with a low peak inspiratory pressure (PIP35 cm H_2_O)  Ventilatory parameters were recorded using a computerized system.  **Level 3** | The percentage of relevant mask leak significantly decreased (p35 cm H2O was 19.5% (SD 32.8%) at t1 and 39.2% (SD 37.7%) at t2 (padj = 0.27; β = +0.61, SE = 0.36) and significantly decreased (padj = 0.01; β = -1.61, SE = 0.55) to 6.0% (SD 15.4%) at t3. | 2c |
| **McDermott et al, 2001**  **Indonesia** | Journal of Midwifery and Women’s Health 46(4):217-25 | | To evaluate Life Saving skill (LSS) in-service training program in Indonesia. The knowledge, confidence and skills of midwives who attended a long, short intensive LSS training program (11-day LSS+ peer review-PR/continuous medical education CME) compared 7-day LSS for midwifery interns and midwives who received no LSS training | Based on the American College of Nurse-Midwives LSS training plus Antenatal care, labour, delivery, post-partum care with a focus on postpartum haemorrhage and neonatal asphyxia | Intern program (7-day LSS training): 28 midwives  LSS+PR/CME (11-day LSS training): 33  Untrained (No LSS training): 47 | **Cross-sectional study**  Evaluators blinded to the participants training status  Self-reported confidence on a scale of 0 (not confident) to 3 (confident) on 50 maternal and neonatal skills  Knowledge test consisted of 26 scenario-based questions (30-40 minutes) covering ANC, labour care, delivery and post-partum care.  Skills assessments: Case scenarios and clinical simulations in infection prevention, use of partograph, manual removal of placenta, bimanual uterine compression and neonatal resuscitation. Minimum level for skills test was 70%  **Levels 1, 2** | **Confidence**:  LSS+PR/CME 62% vs. untrained 57% p=0.14 (No difference)  Internship 65% vs. untrained 57% p=0.12 (No difference)  LSS+PR/CME 62% vs. Internship 65% p=0.51 (No difference)  **Knowledge:**  LSS+PR/CME 65% vs. untrained 59% p=0.006 (significant difference)  Internship 62% vs. untrained 59% p=0.12 (No difference)  LSS+PR/CME 65% vs. Internship 62% p=0.24 (No difference)  **Skills:**  LSS+PR/CME 71% vs. No training 51%, p<0.001 (significant difference)  Interns 62%, and no training 51%, p<0.006 (significant difference)  LSS+PR/CME 71% vs. Interns 62%, p<0.009 (significant difference)  Village midwives from the intensive program scored significantly higher in the practical demonstration of manual removal of placenta, bimanual compression, and neonatal resuscitation than the interns, but the scores on infection prevention and use of the partograph were not different between the two groups. | 3b |
| **Moran et al, 2015**  **South Africa** | Best Practice & Research Clinical Obstetrics and Gynaecology 29(8):1102-18 | | To describe the scale-up of Emergency Obstetric and Newborn Care training in one province in South Africa, including introduction into undergraduate curricula | ESMOE: A derivative of LSTM-RCOG LSS EmOC training, multi-disciplinary, simulation-based ‘skills and drills’ using Training of Trainers approach | >350 ‘Master trainers’, total participants not specified | **Before-after study** with a description of lessons learnt and strategies for improving the effectiveness of scale-up  **Level 2** | EmOC training increases knowledge and skills at an undergraduate level (N=45 pre-test mean score 4.42 SD 1.57, CI (3.95-4.9); post-test mean score 9.11 SD 1.48 CI (8.67-9.56). Introducing EmOC training into undergraduate curricula is feasible. Durability and preparedness following training are dependent on a sufficiently enabling environment. | 2b |
| **Msemo et al, 2013**  **Tanzania** | Pediatrics 131(2):e353-e360 | | To evaluate the impact of the Helping Babies Breath (HBB) program on early neonatal mortality and fresh stillbirth rate | 2-day training of 40 master training using HBB curriculum.  The Master trainers trained healthcare workers from 8 hospitals using 1-day HBB curriculum  Master trainers subsequently provided on-the-job supervision and refresher training | 8 hospitals (3 main referral, 4 regional and 1 district) | **Before-after study**  Before n=8,124, After n=78, 500  Primary outcomes: reduction in early neonatal deaths within 24 hours and fresh stillbirth rate  **Level 4** | Implementation of HBB training was associated with a significant reduction in neonatal deaths (relative risk [RR] 0.53; 95% confidence interval [CI] 0.43-0.65; p≤0.0001) and rates of FSB (RR with training 0.76; 95% CI 0.64-0.90; p=0.001). Stimulation use increased from 47% to 88% (RR 1.87; 95% CI 1.82-1.90; p≤0.0001) while suctioning increased from 15% to 22% (RR 1.40; 95% CI 1.33-1.46; p≤0.0001) whereas face mask ventilation decreased from 8.2% to 5.2% (RR 0.65; 95% CI 0.60-0.72; p≤0.0001). | 2c |
| **Nelissen et al, 2014**  **Tanzania** | Acta Obstetricia et Gynecologica Scandinavica 93(3):287-95 | | To evaluate “Helping Mothers Survive Bleeding After Birth” (HMS BAB) simulation-based training in a low resource setting, a half-day simulation-based training, using a train-the-trainer model | Basic delivery care, active management of the third stage of labour and treatment of postpartum haemorrhage, including bimanual uterine compression | 4 master trainers trained  8 local facilitators  89 healthcare providers (Clinicians 10, Nurse-Midwives 60, Medical Attendants 13, Ambulance drivers 6, involved in maternity care) | **Before-after study** design, using questionnaires information on course perception  Knowledge, skills and conﬁdence of facilitators and learners were tested. 70% or more was considered a pass mark  Skills test was performed one week before and repeated one week after the training  **Levels 1, 2** | After training, all facilitators passed the knowledge test, but pass rates for the skills test were low (29% pass rate for basic delivery and 0% pass rate for management of postpartum haemorrhage).  **Level 1:** HMS BAB training was considered acceptable and feasible, although more time should be allocated for training, and teaching materials should be translated into the local language.  **Level 2**: Knowledge (74% to 80%), skills, and conﬁdence of learners increased signiﬁcantly immediately after training. However, overall pass rates for skills tests of learners after training was low (3% pass rate for basic delivery and management of postpartum haemorrhage). | 2c |
| **Nyamtema et al, 2011**  **Tanzania** | Human Resources for Health 9(1):e28 | | Evaluation training of non-physician clinicians in Emergency Obstetric Care | 3-month hands-on, didactic and clinical training sessions for comprehensive emergency care and anaesthesia for caesarean section Healthcare centres were upgraded | 43 healthcare providers (Assistant medical officers, nurses/midwives and clinical officers) from 12 healthcare centres at the district level in Tanzania and Somalia | **Before-after study**  Outcome measures: Number of institutional deliveries, change in stillbirth rates and obstetric referrals  **Level 4** | During training, participants performed 278 major obstetric surgeries, 141 manual removals of placenta and evacuation of incomplete and septic abortions, and 1161 anaesthetic procedures under supervision. The first 8 months after introduction of CEmOC services in 3 health centers resulted in 179 caesarean section, a remarkable increase of institutional deliveries by up to 300%, decreased fresh stillbirth rate (OR: 0.4; 95% CI: 0.1-1.7) and reduced obstetric referrals (OR: 0.2; 95% CI: 0.1-0.4)). There were two maternal deaths, both arriving in a moribund condition. | 2c |
| **Nyamtema et al, 2016**  **Tanzania** | PLoS ONE 11(3):e0151419 | | To determine the feasibility and impact of decentralizing comprehensive emergency obstetric and neonatal care services in underserved rural areas using associate clinicians. | Complex interventions were introduced at health centers: Physical infrastructure and training.  Supportive supervision and clinical audits were introduced in health centers and district hospitals 2 years after the other interventions were introduced. | 23 assistant medical officers 44 midwives and clinical officers trained in CEmONC and anaesthesia. | **Before-after study**  After interventions were put in place, CEmOC services were launch between 2009 and 2012.  **Level 3** | Mean number of monthly deliveries increased by 151%, obstetric referrals decreased from 9% to 3% (p=0.03) in HCs | 2c |
| **Osei et al, 2005**  **Ghana** | Report  Improving the Ghanaian Safe Motherhood Program: Evaluating the effectiveness of alternative training models and other performance improvement factors on the quality of maternal care and client outcomes  Washington, DC: Frontiers in Reproductive Health, Population Council | | To compare the effectiveness of two methods of Emergency Obstetric Care (EmOC) training: a traditional 3-week residential approach (TRA) versus self-paced learning (SPL) SPL also had component of supportive supervision | Infection prevention practices, management of pregnancy-induced hypertension, anaemia and post-partum haemorrhage, use of the partograph, infant resuscitation, post-abortion care, family planning counselling and methods | 35 midwives in the TRA and 40 in SPL group, from 4 districts (38 healthcare facilities) | **Before-after study** Comparison of impact on knowledge and skills, quality of care and change in provider performance in managing obstetric complications, normal labour and delivery, antenatal and post-natal care; cost comparison is done  **Level 2** | Statistically significant improvement in knowledge scores for the SPL group only.  **Provider performance indicator**: Both approaches showed improvements for all 3 indices (SPL: 10-26%, TRA: 8-22%) but only statistically significant in managing obstetric emergencies by the SPL group (49% baseline, 55% end line) vs. TRA (54% baseline and 58% end line). There was a statistically significant improvement in both groups for labour and delivery skills but no statistically significant difference in PAC skills (n=13)  **Cost-effectiveness:** The SPL approach costs more per trainer than the TRA but the TRA was more cost-effective than the SPL approach across all 3-performance indicators. | 2c |
| **Opiyo et al, 2008**  **Kenya** | PLoS ONE 3(2): e0001599 | | To determine if a simple 1-day newborn resuscitation training (NRT) alters healthcare worker resuscitation practices in a public hospital setting in Kenya  To evaluate the impact of early and late training in newborn resuscitation | Newborn resuscitation based on UK Resuscitation Council guidelines | Early training with NRT (n=28) or late training (the control group, n=55) | **Randomized controlled trial**  Primary outcome: the proportion of appropriate initial resuscitation steps with the frequency of inappropriate practices as a secondary outcome, data collected through direct observation of 97 and 115 resuscitation episodes over 7 weeks after early training in the intervention and control groups respectively.  Baseline routine data were collected 6 months pre-intervention, 3 months after the first training and 3 months after all staff were trained  **Level 3** | Trained providers demonstrated a higher proportion of adequate initial resuscitation steps compared to the control group (trained 66% vs control 27%; risk ratio 2.45, [95% CI 1.75–3.42], p=0.001, adjusted for clustering). There was a statistically significant reduction in the frequency of inappropriate and potentially harmful practices per resuscitation in the trained group (trained 0.53 vs control 0.92; mean difference 0.40, [95% CI 0.13-0.66], p=0.004). | 1b |
| **Rahman et al, 2017**  **Bangladesh** | PLoS ONE 12(10): e0170267 | | Evaluation of complex demand and supply interventions (including training in EmOC) to improve facility delivery rates | **Supply-side interventions**: Upgrading of healthcare facilities, Training in basic delivery and EmOC, 24/7 service provision, improved drugs and supplies, and incentive to healthcare providers.  **Demand-side interventions**: financial incentives, provision of emergency transport, referral support to the tertiary-level hospital. | 6 midwives in 3 primary healthcare facilities, each serving a population of 25,000 | **A before-after observational study conducted over 2 years.**  **Level 3** | 1). Increased facility delivery rate from 25% to 77.6% in 24 months  2) The facility delivery rate was lower when only supply-side interventions were implemented.  3) When financial barriers to accessing facility obstetric care were removed, facility delivery rate increased from 47.1% | 2c |
| **47.**  **Ronsmans et al, 2001**  **Indonesia** | Tropical Medicine and International Health 6(10):799-810 | | To evaluate the impact of Life Saving Skills (LSS) training in Indonesia | American College of Midwives LSS training: normal obstetric care, newborn care and post-natal care.  The intervention comprised of LSS training, interpersonal communication training (IPC), peer review supervision system, continuing education, a maternal and perinatal audit system and an information, education and community strategy aimed at the community | 128 facility-based midwives and 284 village midwives in 3 districts | **Multiple intervention studies**  Quasi-experimental study design: a before-after comparison.  5 skills were assessed. For assessment of IPC, direct observation of practice and client exit interviews were conducted at 3- and 12-months post training. Midwives who had only IPC training were compared to those who had both IPC and LSS training.  **Level 2** | Improvement in the ability of both facility- and village-based midwives to perform the 5 key lifesaving skills. For facility-based midwives, the mean scores increased from 40 to 67% and the percentage ‘competent’ rose from 0 to 46% (p<0.01). For village midwives, mean scores increased from 51 to 71% and the percentage ‘competent’ from 6 to 67% (p<0.01). Training enhanced the IPC skills of village midwives. Mean scores for IPC trained village midwives decreased from 78% at 3 months to 64% at 16 months after training. | 2c |
| **48**.  **Sakeah et al, 2014**  **Ghana** | Reproductive Health 11:90 | | To assess how and to what extent the Community Health Officer (CHO) midwifery program has been integrated into the existing Community-based  Health Planning and Services (CHPS) | 2-year training of community health officers in midwifery skills, including emergency obstetric and newborn care | 10 CHO midwives  6 CHO midwife supervisors | **Qualitative-intrinsic case-study design**  41 in-depth interviews with health professionals and community stakeholders to assess the extent to which the program to train CHOs in midwifery skills has been effective in bridging the gap in skilled birth attendance  **Levels 2, 3** | The CHO-midwives provide integrated services that include skilled delivery in CHPS zones and collaborate with District Assemblies, Non-Governmental Organizations and communities to offer skilled delivery services in rural communities. They refer pregnant women with complications to district hospitals and health centres for care, and there has been observed improvement in the referral system. Stakeholders reported community members’ access to skilled attendants at birth, health education, antenatal attendance and postnatal care in rural communities. |  |
| **49.**  **Singhal et al, 2012**  **Kenya and Pakistan** | Resuscitation 83(1):90-6 | | To evaluate the educational program in neonatal resuscitation for resource-limited settings at facilitator and learner levels | Helping Babies Breathe emphasizes assessment at birth, stimulation to breathe, and assisted ventilation  Use of contemporary educational theory and research and includes evidence-informed content, active learning, skill practice with feedback, case scenarios, self-reflection, group discussion and structured assessment of knowledge, skills and performance | 31 facilitators  102 learners | **Before-after study**  Focus groups provided data on facilitator and learner perceptions used to supplement level K1 data.  K level 2: Knowledge and skill assessments included pre and post scores from multiple choice questions and post-training assessment of bag and mask skills, as well as 2 objective structured clinical evaluations  **Levels 1, 2** | Participants expressed high satisfaction with the program and high self-efficacy with respect to neonatal resuscitation. Assessment of participant knowledge and skills pre and post-program demonstrated significant gains. However, the majority of participants could not demonstrate mastery of bag and mask ventilation on the post-training assessment without additional practice. | 2c |
| **50.**  **Sorensen et al, 2010**  **Tanzania** | International Journal of Gynecology and Obstetrics 111(1):8-12 | | To evaluate the management of prolonged labour and neonatal care before and after Advanced Life Support in Obstetrics (ALSO) training | Major obstetric and perinatal complications:  newborn resuscitation,  estimation of blood loss, active management of the third stage of labour and teamwork | Number of mid and high-level staff providing childbirth trained was not specified | **Prospective before-after study**  558 deliveries before and 550 deliveries after  Outcomes measures: Caesarean section rate, vacuum delivery rate, stillbirth rate, APGAR score and newborn resuscitation  **Level 4** | There was no difference in caesarean section rate, vacuum delivery was not practised after the training, the decision to action time in cases of prolonged labour was more than 3 hours as well as no difference in stillbirth rate, Apgar scores or frequency of newborn resuscitation.  However, there was a significant increase in the number of babies handed to their mothers within 10 minutes 5.6% to 71.5% (RR 12.71; 95% CI, 9.04-17.88). Immediate neonatal mortality decreased as well: 6 deaths before, 0 deaths after p=0.003) | 2c |
| **51.**  **Sorensen et al, 2011**  **Tanzania** | Acta Obstetricia et Gynecologica Scandinavica 90(6):609-14 | | To evaluate the impact of the Advanced Life Support in Obstetrics (ALSO) training on staff performance and incidence of post-partum haemorrhage (PPH) at a regional hospital in Tanzania | Lectures, workshops and case discussions on the major obstetric and perinatal complications: estimation of blood loss, active management of the third stage of labour, teamwork in the step wise management of PPH | 2 medical doctors, 4 medical assistants  16 midwives | **Before-after study** Assessment pre- and 7 weeks post-training on PPH prevention and management estimated blood loss was compared with measured actual blood loss  **Levels 3, 4** | Reduction of incidence of PPH from 32.9 to 18.2% [RR 0.55 (95%CI: 0.44–0.69)] and severe PPH from 9.2 to 4.3% [RR 0.47 (95%CI: 0.29–0.77)]. Staff identiﬁed 1 in 25 PPH cases before and 1 in 5 after training. A signiﬁcantly higher proportion of women with PPH were managed with continuous uterine massage, oxytocin infusion and bimanual compression of the uterus after the training. There was **a** significant decrease **in** episiotomies. | 2c |
| **52.**  **Spitzer et al, 2014**  **Kenya** | International Journal of Gynecology and Obstetrics 127(2):189-93 | | To determine the impact of introducing an Emergency Obstetric and Neonatal Care training program on maternal and perinatal morbidity and mortality at Moi Teaching and referral hospital, Eldoret, Kenya | Advances in Labor and Risk Management International Program (AIP), a 5-day capacity-building course for physicians, physicians-in-training and midwives designed to address the five main causes of maternal mortality and morbidity (obstructed labour, haemorrhage, sepsis, hypertensive disorders, and complications of unsafe abortion) and newborn health outcomes | 80% of health professionals working at Moi Teaching and Referral Hospital from November 2009 to August 2011 | **Before and after study** using prospective chart reviews of all deliveries during the 3-month period before the introduction of the AIP and 3 months period, 1 year after 80% of the staff had been trained in AIP. The primary outcome was the direct obstetric case fatality rate  **Levels 2, 3, 4** | A total of 1741 deliveries occurred during the baseline period and 1812 in the postintervention period. Only one mother died in each period. However, postpartum haemorrhage rates decreased, affecting 59 (3.5%) of 1669 patients before implementation and 40 (2.3%) of 1751 afterwards (*P* = 0.029). The number of patients who received oxytocin increased from 829 (47.6%) to 1669 (92.1%; *P* < 0.001). Additionally, the number of neonates with 5‐minute Apgar scores of less than 5 reduced from 133 (7.7%) of 1717 to 95 (5.4%) of 1745 (*P* = 0.006).  There was a significant reduction (p<0.001) in episiotomy rates 11.2% (192) before and 4.7% (84) after 1 year. There was also a significant reduction in post-partum EWPhaemorrhage from 3.3% (59) to 2.3% (40) p<0.001. | 2b |
| **53.**  **Tang et al, 2016**  **Malawi** | International Journal of Gynecology and Obstetrics 132(2):240-3 | | To evaluate whether a hospital-based mentoring program could significantly increase Emergency Obstetric and Newborn Care (EmONC) knowledge and skills | Two phase mentoring and training program that included individual or small-group EmONC sessions in a skills laboratory | 20 mentors trained who then provided training to 114 participants | **Single-center, single-arm interventional study** Evaluation using pre- and post-intervention written and practical tests with post tests conducted immediately following and 6-months after training  **Level 2** | Significantly increased scores for written/knowledge (n=134; difference 22.9%) and practical/skills (n=125; difference 29.5%, p<0.001) immediately after the training. Similar results when comparing pre-training scores and scores at 6 months post training: written/knowledge (n=111, difference 21% p<0.001), practical/skills (n=103, difference 29.3% p<0.001). However, the immediate post training improvement was not retained at 6 months compared to immediately after the training for knowledge (n=111, difference 3.1%, p<0.001) but skills were retained (n=111, difference 1.7% p<0.054).  The training-of-trainers approach led to significantly higher performance on written and practical tests immediately following training and at six-months overall. Lack of practical opportunities led to a significant reduction in knowledge and skills in some EmONC areas. | 2b |
| **54.**  **Taylor, 2010**  **Ghana** | **Report**  Issues in Essential Obstetric Care:  Report of a Technical meeting of the IAG for Safe Motherhood May 31-June 2, 1995. New York: The Population Council; Family Care International. pp. 45-47 | | To describe the training and evaluation of rural based midwives in emergency obstetric care | 2-week long training including: introduction to maternal mortality, hemorrhage, sepsis, pregnancy induced hypertension, obstructed labor, abortion, antenatal risk assessment, use of the vacuum extraction, adult and newborn resuscitation and repair of episiotomies | 123 nurse-midwives | **Observational study**  Participant self-assessment at the end of every module, follow-up field visits with assessment of new skills performed and observation of clinical practice (episiotomy repair, review of partograph)  **Levels 1, 3** | 24 out of the 123 rural based midwives trained and 4 midwifery programs were visited. Midwifery programs had integrated materials from the training into their pre-service curricula. Procedures such as newborn resuscitation had improved, there were two successful adult resuscitations, episiotomies inspected were of high quality, partographs inspected were properly completed and showed sound clinical management decisions had been taken. | 2c |
| **55.**  **Varghese et al, 2016**  **India** | Global Health: Science and Practice 4(4):582-93 | | To test the feasibility, acceptability and effectiveness of a “skills and drills” intervention to improve emergency obstetric and newborn care | Emergency drills delivered every 2 months and 2-day skills refresher session across 4 sub-districts by independent training team | 4 intervention and 4 comparison facilities. | **Quasi experimental study design**  Before and after knowledge and skills assessments, delivery case sheet reviews and in-depth interviews.  Primary outcomes: improved diagnosis and management of post-partum hemorrhage, pregnancy induced hypertension and birth asphyxia. Secondary outcome measures: knowledge and skills of health providers, acceptability and feasibility of the intervention.  **Levels 2, 3** | 1). Improved post training knowledge scores   - EmOC 49% to 57% (p=0.006) - Newborn care 48% to 56% (p=0.3)   2). Knowledge scores in the comparison facilities were similar but did not improve with time  Skills levels were significantly higher in the intervention group compared to the comparison group   - EmOC skills 55% vs 46% (p<0.001) - Newborn skills 58% vs 48% (p<0.001)   3). There was no significant difference between both groups in the diagnosis and management of emergency obstetric and newborn complications.  4). Barriers to provision of EmOC after training were shortages in nurses and doctors, lack of consistent supply chain for drugs and supplies. | 2c |
| **56.**  **Walker et al, 2012**  **Mexico** | International Journal of Gynecology and Obstetrics  116(2):128-33 | | Evaluation of the PRONTO training course | Two modules 3 months apart covering team work, communication, strategic planning, obstetric hemorrhage, neonatal resuscitation, pre-eclampsia and eclampsia | 68 and 44 healthcare providers (general practitioners, midwives, obstetricians, pediatricians, general surgeons and medical interns) participated in modules 1 and 2 respectively | **Before-after study**  Mixed methodology with 3 main outcome measures: 1) Acceptability 2) Institutional goal achievement, 3) Team improvement  Tools: Pre- and post-test, participants’ knowledge and self-efficacy and program evaluation questionnaire  Data was collected at pre, immediately post training and at 3 months post-training  **Levels 1, 2, 3** | The trainees were highly positive in terms of both acceptability and feasibility of the training. There was an improvement in obstetric hemorrhage (50%-59% overall correct responses) and neonatal resuscitation (33%-40% overall correct responses) knowledge between pre- and post- 3 months. Self-efficacy scores measured pre- and post-3 months improved for obstetric hemorrhage, basic delivery care, and obstetric emergency care. Overall, 31 goals were listed, of which 20 (64.5%) were achieved, 9 (29.0%) were in process, and 2 (6.5%) were associated with no action. | 2c |
| **57.**  **Walker et al, 2014**  **Mexico** | BMC Pregnancy and Childbirth 14:367 | | To describe a process indicator analysis of an evaluation that assessed the impact of a highly-realistic, low-tech simulation-based obstetric and neonatal emergency training  Program (PRONTO) on maternal and neonatal outcomes | PRONTO, a highly-realistic, low-tech simulation-based  obstetric and neonatal emergency training program and team training in emergency obstetric and newborn care | 450 professionals (physicians and nurses) in inter-professional teams in 12 intervention facilities | **Before-after study**  12 matched pairs of hospitals: 12 intervention and 12 control hospitals  Data collection in twelve intervention facilities on process indicators, including pre and post intervention changes in knowledge and self-efficacy of obstetric emergencies and neonatal resuscitation, achievement of strategic planning goals and changes in teamwork scores  Authors performed a longitudinal fixed-effects linear regression model to estimate changes in knowledge and self-efficacy and logistic regression to assess goal achievement  **Levels 1, 2, 3** | Significant increases in knowledge and self-efficacy were noted for both physicians and nurses (p<0.001- 0.009) in all domains. Teamwork scores improved and were maintained over a 3-month period. A mean of 58.8% strategic planning goals per team in each hospital were achieved. There was no association between high goal achievement and knowledge, self-efficacy, proportion of doctors or nurses in training, state, or teamwork score. | 2c |
| **58.**  **Walker D et al, 2015**  **Guatemala** | BMC Medical Education 15:117 | | To assess the effect of a low-technology simulation-based training scheme for obstetric and perinatal emergency management (PRONTO) | PRONTO: low-technology, multi-disciplinary, team simulation-based training package | 207: 121 auxiliary nurses, 80 physicians & professional nurses, 2 ‘other’ in 15 facilities (63% of staff) | **Multi-center, single-arm interventional study**  Pre- and post-test of knowledge and self-efficacy  **Levels 1, 2** | Training improved provider knowledge and self-efficacy. | 2b |
| **59.**  **Walker D et al, 2016**  **Mexico** | Simulation in Healthcare 11(1):106 | | To evaluate the effect of a low-technology simulation-based training scheme for obstetric and perinatal emergency management (PRONTO) on patient outcomes | PRONTO, a highly-realistic, low-tech simulation-based  obstetric and neonatal emergency training program and team training in emergency obstetric and newborn care | From 2010 to 2013  12 hospitals in the intervention arm received PRONTO training. Control hospitals received no intervention. | **Paired matched control trial**  Outcome measures: hospital based neonatal mortality rate, maternal complications and caesarean delivery rate. Fitted mixed-effects negative binomial regression models was used to estimate outcome measures, cumulatively, at intervals measured at 4, 8 and 12 months  **Levels 3, 4** | 1). Reduction of C/S rate cumulatively at 12 months in the intervention compared to control group (21% decrease, P=0.005) and at all 3 intervals (4months: 16% reduction, p=0.02, 8months: 20% decrease, p=0.004, 12months: 20% decrease, p=0.003, p=0.003)  2). No significant impact on maternal complications  3).40% significant reduction in neonatal mortality at 8 months post intervention only. | 2b |
| **60.**  **Walton et al, 2016**  **Guatemala** | International Journal of Gynecology and Obstetrics 132(3):359-64 | | To assess the effect of a low-technology simulation-based training scheme for obstetric and perinatal emergency management (PRONTO) | PRONTO: low-technology, multi-disciplinary, team simulation-based training package | An average of 4.0 doctors, 3.4 nurses and 13.3 auxiliary nurses in each intervention clinic | **Paired cross-sectional birth observation study**  8 intervention clinics, 8 control clinics with direct observation following PRONTO training  **Level 3** | Compared to control clinics, participants in interventions clinics were more likely to implement evidence-based practices, provide patient-centered care and demonstrate teamwork and communication skills. | 2b |
| **61.**  **Xu et al, 2014**  **China** | Resuscitation 85(2):253-9 | | To evaluate the effectiveness of a neonatal resuscitation intervention package at county level hospitals  Components of intervention:  - Training workshops  - Training of national and provincial instructors  - Establishment of hospital resuscitation teams | Neonatal resuscitation training | Eleven intervention and 11 control site hospitals were involved with 97 and 87 obstetricians, pediatricians and midwives respectively | **Cluster randomized controlled trial**  4 intervention and 4 control counties  Data on knowledge, self-confidence and incidence of deaths from birth asphyxia was collected for 2 years post intervention  **Levels 1, 2, 3, 4** | **K L1: Self-reported confidence: Intervention=**57.3%+/-2.5%, control=54.1%+/-8.2. p=<0.001)  **K L2: Knowledge assessments:** 92%+/-12%, control=84%+/-1.5. p=<0.001)  **K L3: Implementation of neonatal resuscitation related protocols:** Intervention=90%, control=55%  **K L4**: Incidence of birth asphyxia decreased from 8.8% to 6.0% (p<0.001) in the intervention counties. Asphyxia related deaths in the delivery room decreased from 27.6 to 5.0 per 100,000 (p=0.076)  The asphyxia rates did not decrease over time in the control counties. | 1b |
| **62.**  **Zaeem-ul-Haq et al, 2009**  **Pakistan** | Journal of the Pakistan Medical Association 59(1):22-6 | | To assess motivational level post-training and the extent to which the health providers were using the skills learned | 2 to 5-day Essential Surgical Skills -Emergency Maternal and Child Health (ESS-EMCH) training package; 12 modules - complications of pregnancy, newborn and adult resuscitation, transportation of ill patients, care of the newborn and pediatric emergencies | 234 healthcare providers | **Retrospective cohort study, postal survey**  **Levels 1, 3** | 90% of respondents reported using acquired skills and the structured Airway, Breathing, Circulation (ABC) approach in handling emergencies. Lack of equipment and lack of support from higher levels were common barriers to improving practice. 81% of respondents reported that training had resulted in better availability or use of supplies at their facilities. Healthcare providers caring for neonates expressed having confidence in obstetric procedures, while  obstetrician-gynecologists described themselves as being more confident in neonatal resuscitation. | 2c |
| **63.**  **Zafar et al, 2009**  **Pakistan** | Resuscitation 80(4):449-52 | | To evaluate the use of a structured training program in emergency care in Pakistan through the completion of logbooks documenting actual resuscitation attempts | Essential Surgical Skills-Emergency Maternal and Child Healthcare (ESS-EMCH) training package | 120 healthcare providers | **Observational study** Logbooks to document the use of newly acquired resuscitation skills  **Levels 3, 4** | 1,123 resuscitation attempts were documented by 63 of 120 trained healthcare providers (response rate 53%; number of attempts 4–22 per participant). 76% (858/1123) of documented cases were received from doctors and 24% (265) from nurses. The patients receiving resuscitation were neonates 31% (n=349), infants and children 38% (n=426), pregnant mothers 21% (n=233) and other adults 10% (n=111). Survival rate in cases reported was 89%. | 2b |
| **High-Income Countries** | | | | | | | | |
| **64.**  **Birch et al, 2007**  **United Kingdom** | | Nurse Education Today 27(8):915-22 | To compare the effectiveness of lecture-based teaching (LBT), simulation-based teaching (SBT) or a combination of both types of teaching (LAS) | 1-day Management of Postpartum Hemorrhage training | 6 teams of in total 36 midwives and medical doctors | **Quasi-experimental cohort study**  Assessment pre- and post-training at 3 weeks, 3 months  **Level 2** | Knowledge scores increased by 98, 74 and 75 points for SBL, LAS and LBT respectively. At 3-months post-training the SBT group continued to improve by a further 25 points, LBT declined by 3 points LAS group declined by 4 points (p=0.086). | 2b |
| **65.**  **Burstein et al, 2016**  **USA** | | Health Services Research 51(S3):2472-86 | To establish multifactorial shoulder dystocia response and management  protocol to promote sustainable practice change. | Shoulder dystocia training and protocol implementation | Five health labor and delivery facilities (70 obstetricians, family practice physicians and midwives) | **Case study evaluation** methodology was used to examine clinician engagement and protocol adoption over 2 years  I**ntervention**: Adoption of Ascension Health Shoulder Dystocia Response and Management   - ACOG definition - Tracking of delivery process - Use of one of several man oeuvres - 3 minute time limit - Move to urgent abdominal rescue after 3 minutes - Record time of delivery of body   1.5-hour e-learning module and repeated biennially.  TeamSTEPPS teamwork and communication training  Multidisciplinary team simulation training using high fidelity simulator.  **Level 3** | Over 1 year, 224 obstetric practitioners, mid-level providers and 349 nurses were trained (e-learning and protocol).  243 obstetric practitioners and 414 nurses completed the simulation training  1) Improved identification of shoulder dystocia: 99% compliance of SD reporting, representing a 3-fold increase in the first year.  2) 98 percent completed all training elements and in subsequent years, 98% completed the follow-up training.  3) Overall the shoulder dystocia protocol was adopted by 99% of the teams trained | 3 |
| **66.**  **Crofts et al, 2006**  **United Kingdom** | | Obstetrics and Gynecology 108(6):1477-85 | To compare the effectiveness of training with low- and high-fidelity mannequins for the management of shoulder dystocia | Management of shoulder dystocia, 40- minute training | 144 midwives and doctors | **Randomized controlled trial**  2 arms of 72 each  **Level 3** | For both arms, training was associated with improved performance scores for use of basic maneuvers (p<0.002) and successful delivery (p<0.001), good communication with the patient (p<0.001) after training. Training with a high-fidelity mannequin was associated with a higher successful delivery rate than training with traditional devices: 94% compared with 72% (OR 6.53, 95% CI 2.05–20.81). | 1b |
| **67.**  **Crofts, Bartlett et al, 2007**  **United Kingdom** | | Obstetrics & Gynecology 110(5):1069-74 | To assess skills retention 6- and 12-months after shoulder dystocia training | Management of shoulder dystocia, 40- minute training | 122 midwives and doctors from 6 hospitals | **Before-after training**  Participants managed a standardized simulation in shoulder dystocia pre-training and 3 weeks, 6, 12 months post-training  **Level 2** | Before training, 49% achieved delivery. This was 82% after 3 weeks, 84% after 6 months 85% after 12 months. Of 21 providers who could not deliver 3 weeks after training, additional training resulted in 79% achieving delivery at 12 months. | 2b |
| **68.**  **Crofts, Ellis et al, 2007**  **United Kingdom** | | BJOG: An International Journal of Obstetrics and Gynecology 114(12):1534-41 | To explore the effect of obstetric emergency training on knowledge and to assess if acquisition of knowledge is influenced by the training setting or teamwork training | 1- or 2-day course; basic life support, advanced life support, hypertensive disorders of pregnancy, shoulder dystocia, breech, twins, cord prolapse, postpartum hemorrhage and electronic fetal monitoring | 140 participants | **Randomized controlled trial**  Four arms: a) 1-day course at local hospital b) 1-day course at simulation center, c) 2-day course with teamwork training at local hospital d) 2-day course with teamwork training at simulation center  **Level 2** | Mean knowledge score increased by 20.6 points (95% CI 18.1–23.1, p<0.001). Overall, 123/133 (92.5%) participants increased their score. No effect on knowledge score on location of training or inclusion of teamwork training. | 1b |
| **69.**  **Crofts et al, 2008**  **United Kingdom** | | Quality and Safety in Health Care 17(1):20-4 | To explore the effect of training on patient actor perception of care during simulated obstetric emergencies | (As above)  Eclampsia and PPH management assessed only | Midwives and doctors from 6 hospitals | **Randomized controlled trial**  Four arms: a) 1-day course at local hospital, b) 1-day course at simulation center, c) 2-day course with teamwork training at local hospital d) 2-day course with teamwork training at simulation center  **Level 2** | For simulated PPH management, safety and communication scores were higher when patient-actor was cared for by teams trained locally with a patient-actor compared with teams trained at the simulation center using a computerized patient mannequin (safety p=0.048, communication p=0.035), with a trend towards higher scores for respect (p=0.077). For eclampsia this was similar with a trend towards better communication scores for teams trained with patient-actors compared to using mannequins (p=0.071). Additional teamwork training did not influence scores significantly. | 1b |
| **70.**  **Crofts et al, 2013**  **United Kingdom** | | International Journal of Gynecology and Obstetrics 123(1):81-5 | To determine knowledge retention 1-year after training for intrapartum emergencies (including shoulder dystocia, eclampsia, and postpartum hemorrhage)  Methods: Training was undertaken in 6 hospitals and the Bristol Medical Simulation Centre, UK | 1-day clinical training course on obstetric emergencies at a local hospital; or a 2-day clinical and teamwork training course on obstetric emergencies at a simulation center with or without additional teamwork training | Participants (22 junior and 23 senior doctors-obstetricians and anesthetists, 47 junior and 48 senior midwives) | **Randomized controlled trial**  The primary outcome was change in factual knowledge over time, as assessed by a 185-question multiple-choice questionnaire and simulated obstetric emergency (shoulder dystocia, eclampsia, and postpartum hemorrhage)  Participants underwent a baseline assessment before attending their training course. The assessment was repeated at 3 weeks, 6 months and 12 months after training  **Level 2** | Mean scores at 6 (97.6 ± 23.0; n=107) and 12 (98.2 ± 21.6; n=98) months remained higher than those before training (79.6 ± 21.9, n=140; both p<0.001), but were slightly lower than those immediately after training (101.0 ± 21.3, n=133; p<0.001 and p=0.007, respectively). The type of training had no effect on retention of knowledge. | 1b |
| **71.**  **Daniels et al, 2010**  **USA** | | Simulation in Healthcare 5(1):40-45 | To determine whether simulation was more effective than traditional didactic instruction to train crisis management skills to labor and delivery teams | 3-hour training in shoulder dystocia and eclampsia | 32 doctors and midwives, in teams of 4 | **Randomized controlled trial**  2 arms: didactic training (DT) or simulation-based training (SBT), before and after evaluation of knowledge and performance, 1-month post training  **Level 2** | No statistical difference in knowledge between groups. For both shoulder dystocia and eclampsia drills, SBT scored higher than DT (p=0.002 and p=0.32, respectively) | 1b |
| **72.**  **Deering et al, 2004**  **USA** | | Obstetrics and Gynecology 2004; 103:1224-8 | To determine whether a simulation training scenario improves resident competency in the management of shoulder dystocia. | Management of shoulder dystocia. | 33 residents (16 trained, 17 untrained) | **Randomized controlled trial**  Residents from 2 training programs participated in this study.  The residents were block-randomized by year-group to a training session on shoulder dystocia management that used an obstetric birthing simulator or to a control group with no specific training.  Trained residents and control subjects were subsequently tested on a standardized shoulder dystocia scenario, that were digitally recorded.  An independent external assessor graded and rated the resident's performance with a standardized evaluation sheet.  Statistical analysis included the Student *t* test, [chi]^2^, and regression analysis, as appropriate.  **Level 3** | 1). Trained residents had significantly higher scores in all evaluation categories, including timelines of their interventions, performance of maneuvers, and overall performance.  2). They also performed the delivery in a shorter time than control subjects (61 versus 146 seconds, P .003). | 1 |
| **73.**  **Dijkman et al, 2010**  **Netherlands** | | BJOG: An International Journal of Obstetrics and Gynaecology 117(3):282-7 | To determine the incidence of a peri-mortem caesarean section (PMCS) after introduction of the Managing Obstetric Emergencies and Trauma (MOET) course | 3 -day training in (MOET) | 55 women had cardiac arrest and 12 of them underwent PMCS | **Secondary data analysis**  Retrospective cohort study, 15-year period  **Levels 3, 4** | Before the introduction of the MOET course, 4 PMCS were performed (0.36/year), compared to 8 PMCS after (1.6/year, p=0.01).  No PMCS was performed within the recommended 5 minutes after starting resuscitation. 8 of the 12 women regained cardiac output after PMCS, with two maternal and five neonatal survivors. | 2c |
| **74.**  **Draycott and Crofts, 2006**  **United Kingdom** | | Fetal and Maternal Medicine Review 17(3):229-37 | To determine whether the introduction of obstetrics  emergency training  was associated with  a reduction in perinatal asphyxia and neonatal hypoxic–ischemic encephalopathy | 1-day training; cardiotocography Interpretation, 6 obstetric emergency  drill stations; shoulder dystocia, postpartum  hemorrhage, eclampsia, twins, breech, adult resuscitation and neonatal resuscitation | Not specified | **Secondary data analysis**  Retrospective cohort study of babies born between 1998 and 2003, training introduced in 2000;  19,460 infants included  **Level 4** | Following the introduction of training, the number of babies born with 5-minute Apgar scores of <6 decreased from 86.6 to 44.6 per 10,000 births (p<0.001). HIE decreased from 27.3 to 13.6 per 10,000 births (p=0.032) Stillbirth rates remained unchanged. | 2c |
| **75.**  **Egenberg et al, 2015**  **Norway** | | Acta Obstetricia et Gynecologica Scandinavica 94(3):316-23 | To investigate whether inter-professional simulation training influenced the rate of red blood cell transfusions after birth in a Norwegian university hospital | A 6-hour scenario-based training on emergency obstetrics including postpartum hemorrhage, using a birthing stimulator | All maternity staff at the university hospital | A **pre-post design- retrospective comparison** of two cohorts of women with estimated blood loss >500ml within 24 hours after birth  Main outcome measure was the frequency of women receiving red blood cell transfusions as a marker for blood loss  Secondary measures- frequency of surgical procedures in the management of postpartum hemorrhage  **Levels 3, 4** | There was a significant reduction in the number of women receiving RBC transfusion after birth post-intervention, compared with before the intervention: 2009, 111/534 (20.8%) vs 67/546 (12.3%) in 2011 received blood transfusions. The odd ratios 2011 vs 2009 was 0.53 (95% CI 0.38-0.74) as well as a significant reduction in frequencies of curettage (p<0.01) and uterine artery embolization (p=0.01) after the intervention | 2b |
| **76.**  **Ellis et al, 2008**  **United Kingdom** | | Obstetrics and Gynecology 111(3):723-31 | To compare the effectiveness of training for  eclampsia in local hospitals and a regional simulation  center, with and without teamwork training | Management of eclampsia | 24 teams of midwives and doctors | **Randomized controlled trial**  4 arms: training in local hospital or in a simulation center and teamwork training or not  **Level 4** | Training was associated with an increase in completion of basic tasks; 87% before and 100% after training. Basic tasks were completed more quickly (p=0.012), magnesium  Sulphate loading dose was administered by 61% of teams before and 92% after training (p=0.04), median time to administration decreased (p=0.011). Training at the simulation center was not associated with additional improvement compared to training in the hospital setting. Teamwork  improved overall after training (p<0.001) with no additional benefit of teamwork training per se. | 1b |
| **77.**  **Grobman et al, 2011**  **USA** | | American Journal of Obstetrics & Gynecology  205(6):513-7 | The objective of this study was to assess outcomes that are associated with the implementation of a shoulder dystocia protocol that is focused on team response. | Training and implementation of a shoulder dystocia protocol | All care providers on the labor delivery unit. Specific number not specified. | **Before-after training**  Women who had a shoulder dystocia during 3 time periods: 6 months before (period A), 6 months during (period B), and 6 months after (period C) the institution of a shoulder dystocia protocol, were identified.  Documentation and health outcomes were compared among the time periods  **Level 4** | 1). During the study period, 254 women (77, 100, and 77 in periods A, B, and C, respectively) had a shoulder dystocia.  2). There were no differences among study periods in patient characteristics.  3). Complete and consistent documentation increased (14% to 50% to 92%; P < .001)  4). Brachial plexus palsy decreased: diagnosed at delivery (10.1% to 4.0% to 2.6%; P <.03) and at neonatal discharge (7.6% to 3.0% to 1.3%; P <.04. |  |
| **78**  **Inglis et al, 2011**  **USA** | | American Journal of Obstetrics & Gynecology  204:322.e1-6. | To determine whether implementation of shoulder dystocia training reduces the incidence of obstetric brachial plexus injury (OBPI) | Mandatory hands-on simulated shoulder dystocia management training was instituted during July and August 2006  and proficiency with the protocol was tested with a practical examination. Unsatisfactory proficiency warranted repeat training sessions and practical examination. | All maternity staff  all hospital labor and delivery staff (attending physicians, resident physicians, midwives, and nurses) | A retrospective cohort study was conducted using information gathered from electronic medical records of mothers and their infants born from Aug. 1, 2003, through Dec. 31, 2009.  All shoulder dystocia cases before July 1, 2006, were considered to be pretraining cases and all shoulder dystocia cases after Aug. 31, 2006, were considered to be post-training cases.  **Level 4** | 1). The overall incidence of OBPI in vaginal deliveries decreased from 0.40% pre-training to 0.14% post-training (P <.01).  2). OBPI after shoulder dystocia dropped from 30% to 10.67% post-training (P < .01). Maternal body mass index (P < .01) and neonatal weight (P= .02) decreased and head-to-body delivery interval increased in the post-training period (P= .03).  3). Only shoulder dystocia training remained associated with reduced OBPI (P= .02) after logistic regression analysis.  4). OBPI remained less in the post-training period (P= .01), even after excluding all neonates with birthweights >2 SD above the mean. |  |
| **79.**  **Fisher et al, 2010**  **USA** | | American Journal of Obstetrics & Gynecology 203(4):379,e1-5 | To compare eclampsia and magnesium toxicity management among residents randomly assigned to lecture- or simulation-based education | Eclampsia and magnesium toxicity management | 38 residents in obstetrics and gynecology | **Randomized controlled trial**  3 arms: Simulation and Lecture (SL), Simulation (S), and Lecture (L)  **Level 2** | Post training maternal scores were significantly better in S compared with L groups (p<0.05). Post intervention magnesium toxicity and fetal scores were not different between groups. Adding lectures to simulation training (SL) did not lead to an incremental benefit. | 1b |
| **80.**  **Fransen et al, 2012**  **Netherlands** | | BJOG: An International Journal of Obstetrics & Gynaecology 119(11):1387-93 | To determine whether obstetric team training in a medical simulation improves the team performance and utilization of appropriate medical technical skills of healthcare professionals | Staff from 24 obstetric departments was randomly assigned to a 1-day session of multi-professional team training in a medical simulation Centre (80% crew resource training and 20% medical technical skills) or to no such training  The Team training was given with high-fidelity mannequins by an obstetrician and a communication expert | The obstetric departments of 24 Dutch hospitals  12 hospitals with 74 obstetric teams in the intervention group | **Cluster randomized control trial**  Team performance evaluated after 6 months with the validated Clinical Teamwork Scale (CTS) and the employment of two specific obstetric procedures for the two clinical scenarios in the simulation (delivery of the baby with shoulder dystocia in the maternal all-fours position and conducting a peri-mortem caesarean section within 5 minutes for the scenario of amniotic fluid embolism).  Carried out during unannounced sessions that were videotaped. Two independent experts evaluated the team performance and utilization of appropriate medical skills.  **Level 3** | Teamwork performance in the training group (74 obstetric teams from 12 hospitals) was significantly better in comparison to the non-training group (median CTS score: 7.5 versus 6.0, respectively; p=0.014). The use of the predefined obstetric procedures for the two clinical scenarios was also significantly more frequent in the training group compared with the non-training group (83 versus 46%, respectively; p=0.009).  Conclusions: Team performance and medical technical skills may be significantly improved after multi-professional obstetric team training in a medical simulation center. | 1b |
| **81.**  **Freeth et al, 2009**  **United Kingdom** | | Journal of Continuing Education in the Health Professions 29(2):98-104 | To describe and evaluate the Multi-disciplinary Obstetric Simulated Emergency Scenarios course (MOSES), which was designed to enhance non-technical skills among obstetric teams and, evaluate participants’ perceptions, their learning and the transfer of learning to clinical practice | 1- day MOSES course | Thirteen MOSES courses were conducted with 93 course participants comprised 57 midwives, 21 obstetricians, and 15 anesthetists. 55 (59%) participants contributed to this evaluation | **Prospective cohort study**  Modified Kirkpatrick evaluation method. E-mail and telephone interviews. Facilitator feedback was received from 9 courses, spanning the 4 DS teams. Video recordings of sufficient quality for analysis were received from 11 courses, spanning 3 delivery suite (DS) teams.  **Levels 1, 3** | The team training was well received. Participants were able to check out assumptions and expectations of others and develop respect for different roles within the delivery suite team. Skillful facilitation of debriefing after each scenario was central to learning. Participants reported acquiring new knowledge or insights, particularly concerning the role of communication and leadership in crisis situations. | 2b |
| **82.**  **Gardner and Raemer, 2008**  **USA** | | Obstetrics and Gynecology Clinics of North America 35(1):97-127 | To describe the development, implementation and evaluation of an obstetric simulation-based team training course grounded in crisis resource management principles | Obstetric emergencies, simulation-based team training | Pilot course: 36 healthcare providers, Obstetric simulation-based team training: 176 participants | **Cohort study**  Trainee course evaluation immediately after pilot course and 1-year or more post training follow-up with self-assessment questionnaires.  **Levels 2, 3** | The course was highly regarded immediately and 1 year or more after completing the course. Most survey responders reported improved teamwork and communication when managing a critical obstetric event since training. | 2b |
| **83.**  **Guise and Segal, 2008**  **USA** | | Best Practice and Research: Clinical Obstetrics and Gynaecology 22(5):937-51 | To determine the added value of in-situ obstetric simulation training over training at a simulation center | In situ obstetric simulation training: shoulder dystocia, postpartum hemorrhage, eclampsia | 150 healthcare workers (nurses and medical doctors) from labor wards across 6 hospitals | **Before-after training**  comparison with short debrief after each scenario and didactic session  **Level 1** | Participants reported that in situ simulation was more appropriate and cost effective. Conducting simulation in the location where care is delivered is convenient for staff and maximizes learning because the participants are in a familiar environment. This approach is also considered useful to explore and analyze system issues that affect quality of care delivered during emergencies. | 2b |
| **84.**  **Harris et al, 1995**  **USA** | | Journal of Obstetric, Gynecologic, and Neonatal Nursing 24(9):829-35 | To determine whether there was an increase in knowledge when a multi-disciplinary group of healthcare providers participated in the same comprehensive perinatal continuing education program and to determine whether care practices followed before the program differed from those followed 1 year after its completion | Perinatal Continuing Education Program: emergency obstetric and newborn complications including endotracheal intubation and umbilical catheter insertion | Physicians, nurses, and other providers of perinatal health care  Hospitals in Oklahoma providing perinatal care  Test data derived from healthcare providers in 24 hospitals; data on care practices obtained from 12 hospitals | **Cohort study**  Pre-and post-test knowledge assessment and 1-year post training, audit of medical records of sick or at risk newborns  **Levels 2, 3** | Pre- and post-training knowledge scores show increase for each group of providers studied (p<0.001). Statistically significant increases in use of 2 care practices and a trend toward an increase in the use of 3 others. Healthcare practices performed significantly more often after training include gestational assessment using the Dubowitz scale taught in PCEP (p=0.001) or any other method (p=0.001) and blood pressure measurement (p=0.003). | 2b |
| **85.**  **Howie, 2011**  **United Kingdom** | | **Report**  Published by the School of Health Nursing and Midwifery  University of the West of Scotland  An evaluation of the Scottish Multi-professional Maternity Development Programme (SMMDP) | To measure the impact on maternity services following the introduction of (SMMDP) | Obstetric and newborn emergencies: normal pregnancy, maternal and newborn resuscitation, stabilization and transfer, uterine rupture and inversion, sepsis, shoulder dystocia, malpresentations, pre-eclampsia/eclampsia, hemorrhage and cord prolapse | Phase 1: NA  Phase 2: 540 participants  Phase 3: 15 | **Retrospective study**  Observational mixed methods study based on Kirkpatrick framework for training evaluation  Phase 1: analysis of pre-existing SMMDP internal course evaluation  Phase 2: evaluation individual course participant’s perception of impact on practice and benefit of the training  Phase 3: evaluation of the perception of impact on practice and cost benefit  **Levels 1, 2** | Phase 1: There was a 90% positive rating on teaching methods, teaching and learning materials, instructor’s helpfulness and knowledge, appropriateness of assessments.  Phase 2: Participants identified if training had increased confidence levels (42%), preparedness for role (37%), knowledge (35%) or level of clinical competence (35%). Overall, participants found the course to be enjoyable, beneficial and an effective mode of training.  Phase 3: The SMMDP was perceived to be cost effective, value for money and an efficient use of time. | 2b |
| **86.**  **MacKenzie et al, 2007**  **United Kingdom** | | BJOG: An International Journal of Obstetrics and Gynaecology 109(6):1059-68 | To investigate trends in the incidence of shoulder dystocia, methods used to overcome the obstruction, and rates of maternal and neonatal morbidity. | Shoulder dystocia simulation training was introduced in 2001 | No specified | **Retrospective data analysis**  Cases of shoulder dystocia and of neonatal brachial plexus injury occurring from 1991 to 2005 were identified.  Management of the shoulder dystocia was recorded.  Demographic data, labor management with outcome, and neonatal outcome were also recorded for all vaginal deliveries over the same period.  **Outcome measures**: Incidence rates of shoulder dystocia and associated morbidity related to the methods used for overcoming the obstruction to labor were determined.  **Level 4** | There were 514 cases of shoulder dystocia among 79,781 (0.6%) vaginal deliveries with 44 cases of neonatal brachial plexus injury and 36 asphyxiated neonates; two neonates with cerebral palsy died.  The McRoberts’ maneuver was used increasingly to overcome the obstruction, from 3% during the first 5 years to 91% during the last 5 years.  The incidence of shoulder dystocia, brachial plexus injury, and neonatal asphyxia all increased over the study period without change in maternal morbidity frequency.  Introduction of the McRoberts’ maneuver did not improved outcomes compared with the earlier results | 2 |
| **87.**  **Markova et al, 2012**  **Denmark** | | Acta Obstetricia et Gynecologica Scandinavica 91(3):346-52 | To evaluate the effect of multi-professional obstetric skills training on the incidence of post-partum hemorrhage (PPH) | Multi-disciplinary emergency obstetric care training shoulder dystocia, PPH, severe pre-eclampsia and newborn resuscitation | 168 Midwives, nurses, auxiliary nurses and doctors on call | **Secondary analysis of data**  Database audit  Information on 10, 461 women were audited, 201 transfusion episodes occurred, of which 179 were included.  **Levels 1, 3 and 4** | There was a significant increase in self-assessed confidence in the management of PPH, improved use of uterotonics and PPH protocols and self-directed infrastructural changes in delivery room such as emergency box for PPH management. There was no significant difference in the rates or requirements for blood transfusion before, during or after the training. There was also no delay in implementing interventions to minimize hemorrhage across all three periods (MVA, MRP or Caesarean section). | 3b |
| **88.**  **Marshall et al, 2015**  **United States** | | Journal of Maternal-Fetal & Neonatal Medicine 28(5):495-9 | To evaluate in-situ simulation and team training for post-partum hemorrhage (PPH) among experienced clinical teams in non-academic hospitals in urban and rural communities | Simulation-based and didactic training on team communication and medical management of PPH | 22 clinical teams from 6 hospitals | **Before-after training**  Simulations of management of PPH before and after a didactic and video training on team communication and medical management of PPH  The simulation scenario was repeated at 9- and 12-months post training.  **Levels 1, 2, 3, 4** | Team training significantly improved response times in the management of postpartum hemorrhage; medical management of PPH improved after training | 2b |
| **89.**  **Maslovitz et al, 2007**  **Israel** | | Obstetrics and Gynecology 109(6):1295-300 | To develop a simulation-based curricular unit for labor and delivery teams involved in obstetric emergencies to detect and address common mistakes | Simulation-based curricular unit for hands-on training of four obstetric emergency scenarios (eclamptic seizure, postpartum hemorrhage, shoulder dystocia, and breech extraction) was developed using high-tech mannequins and low-tech simulators. | 60 residents in obstetrics/ gynecology and 88 midwives.  Obstetric teams consisted of at least one resident and two midwives | **Quasi experimental**  Checklists of actions expected from the teams used for the evaluation by experienced tutors.  All sessions were videotaped and then reviewed and analyzed by the trainees themselves, guided by two experienced tutors.  **Outcome measure**: Most commonly occurring mistakes by summing up checklists and by watching the recorded sessions.  **Level 2** | Forty-two labor and delivery teams completed all four sessions.  The most common management errors were   - delay in transporting the bleeding patient to the operating room (82%), - unfamiliarity with prostaglandin administration to reverse uterine atony (82%), - poor cardiopulmonary resuscitation techniques (80%), - inadequate documentation of shoulder dystocia (80%), - delayed administration of blood products to reverse consumption coagulopathy (66%), - inappropriate avoidance of episiotomy in shoulder dystocia and breech extraction (32%).   18 trainees were invited for repeated sessions at least 6 months after the initial training, and their scores were significantly higher in the latter sessions (79.4 ±4.3 versus 70 ± 5.3 for the second and first simulated eclampsia sessions). | 3 |
| **90.**  **Monod et al, 2014**  **Switzerland** | | Archives of Gynecology and Obstetrics 289(4):733-8 | To investigate the influence of simulation training on four specific skills: self-confidence, handling of emergency situations, knowledge of algorithms and team communication after training in obstetric emergencies | Obstetric emergency training: shoulder dystocia, postpartum hemorrhage, pre-eclampsia, maternal basic life support neonatal resuscitation and operative vaginal birth using low and high-fidelity mannequins | 168 medical doctors and midwives | **Before-after training**  Anonymous self-administered questionnaire with a 5-point Likert’s scale immediately after the training and at 3-months post training.  **Levels 1, 3** | 92.9% (156) response rate immediately after the training but only 36.3% at 3-months. High scores for all 4 skills tested except for communication at 3-months post study p<0.05. | 2c |
| **91.**  **Kim et al, 2016**  **South Korea** | | Nurse Education Today 37(Feb):45-52 | To identify the effects of nursing process-based simulation on knowledge, attitudes and skills for maternal and child emergency nursing care between the experimental and control groups | Nursing process-based simulation training program for the experimental group while the control group received traditional methods of training for maternal and child emergency nursing care. | 49 nurses (25 in the experimental group and 24 in the control group) | **Equivalent control group pre- and post-test experimental design**  **Levels 1, 3** | 1). The experimental group was more likely to improve knowledge, attitudes, and skills about maternal and child emergency nursing care clinical judgement, than the control group.  2). The experimental group was more likely to improve clinical skills required for nursing diagnosis and nursing evaluation than the control group, in all 5 stages of nursing process in simulation. | 2b |
| **92.**  **Nielsen et al, 2007**  **USA** | | Obstetrics and Gynecology 109(1):48-55 | To evaluate the effect of teamwork training on the occurrence of adverse outcomes and process of care in labor and delivery | Standardized teamwork training curriculum based on crew resource management that emphasized communication and team structure and a curriculum used in hospital emergency and obstetric departments | 1,307 labor and delivery room personnel | **Cluster randomized controlled trial**  7 intervention and 8 control hospitals  The primary outcome was the proportion of deliveries at 20 weeks or more of gestation in which one or more adverse maternal or neonatal outcomes or both occurred (Adverse  Outcome Index)  Additional outcomes included 11 clinical process measures  **Levels 3, 4** | The mean adverse outcome Index (range) was 9.4 (6.5-16.6) in the control arm and 9.0 (5.9-14.7) in the intervention arm. One process measure, the time from the decision to perform an immediate caesarean section to the incision, differed significantly after team training (33.3 minutes versus 21.2 minutes, p=0.03). | 1b |
| **93.**  **Noblot et al, 2015**  **France** | | European Journal of Obstetrics & Gynecology and Reproductive Biology 189(2015):101-5 | To evaluate the effectiveness of an inter-disciplinary team training program based on simulated scenarios and focusing on two obstetrical emergency situations | Shoulder dystocia and complicated breech vaginal delivery (CBVD) | 298 healthcare professions (obstetricians, residents in obstetrics, midwives and nursery nurses) | **Before-after study**  Pre-established grids were used to evaluate filmed scenarios managed by teams of 2-3 participants  **Level 2** | 755 (298) of target population were trained over 9 months and there was a significant increase in post training scores 74.5% to 91.4% for shoulder dystocia (p<0.0001) and from 67.2% to 88.4% (p<0.0001) for CVBD. | 2b |
| **94.**  **Ortner et al, 2014**  **USA** | | International Journal of Obstetric Anesthesia 23(4):341-7 | To assess the change in skills following simulation-based training in general anesthesia for caesarean section | During an 8-week obstetric rotation lectures and simulation-based training in anesthesia and emergency caesarean section was delivered | 24 Obstetric residents  Six obstetric anesthesia attending physicians | **Before-after comparative study**  Performance validated assessments were made during the first (pre-test), fifth (post-test) and 8 months post training (post-retention)  Six obstetric attending physicians were assessed using similar scenarios to generate a mean attendings’ performance score  **Level 2** | After one week of training, residents’ performance was significantly below mean, attendings’ performance score (pre-test:159+/-22 vs 159+/11, p=0.013) and remained at that level at eight months (post retention test:164+/-16) | 2b |
| **95.**  **Reynolds et al, 2011**  **Portugal** | | European Journal of Obstetrics & Gynecology and Reproductive Biology 159(1):72-6 | To evaluate the self-perceived impact of attending a simulation-based training course on the management of real-life obstetrical emergencies | Simulation-based training course for the management of four obstetric emergencies | Obstetric nurses and obstetricians (n = 54) from a tertiary care university hospital | **A prospective follow-up study**  One year after the last session of the course, participants were asked to complete a questionnaire evaluating the self-perceived impact it had on their knowledge, technical skills, and teamwork skills during experienced real-life situations using a five-point Likert grading scale  **Level 3** | There was a response rate of 85% (46) and 87% perceived an improvement in their knowledge and skills during real emergencies. Obstetric nurses expressed a significantly higher improvement than obstetricians in their ability to diagnose or be aware of obstetrical emergencies (p=0.002), in their technical skills (p=0.024), and in their ability to deal with teamwork related issues (p=0.005). Participants who had experienced in real-life situations all four simulated scenarios rated the impact of training significantly higher than others (p=0.049) and reported a better improvement in their knowledge of management guidelines (p=0.006). | 2c |
| **96.**  **Robertson et al, 2009**  **USA** | | Simulation in Healthcare 4(2):77-83 | To evaluate a simulation-based team-training program called Obstetric Crisis Team Training Program (based upon the original training program of Crisis Team Training) framed within a multi-level team theoretical model | Training consisted of participation in four standardized, simulated crisis scenarios with a female birthing simulator mannequin: fetal bradycardia, anaphylactic shock, shoulder Dystocia and postpartum hemorrhage | 22 perinatal healthcare professionals (attending medical doctors, nurses, residents, and nurse- midwives) | **Quasi-experimental, before-after comparison**  **Levels 1, 3** | Significant improvement in 3 outcome variables (p<0.004); attitude toward competence in handling obstetric emergencies (t=1.6), individual (t =4.2) and team performance (t=4.1). The remaining 6 variables; attitude toward simulation technology, attitude toward rapid response team, confidence in handling obstetric emergencies, utility of team skills in the workplace, comfort in assuming various team roles, and knowledge, were not statistically significantly different. Overall task completion from the first to the last simulation substantially improved (p<0.05). | 2c |
| **97.**  **Siassakos et al, 2009**  **United Kingdom** | | Journal of Obstetrics and Gynecology 29(6):499-503 | To assess the validity of mixed techniques for the analysis of team communication and whether clinical and non-clinical team training improve communication | All teams received emergency obstetric clinical training (TW-) but only two out of four teams received additional aviation-based crew resource management teamwork training (TW+): roles and responsibilities, clear directed communication, situational awareness | 24: 4 teams of 6 medical doctors and midwives | **Randomized controlled trial**  2 teams in the intervention and control groups each.  **Level 3** | Proportion of directed commands higher after training for teams that received additional teamwork training (TW+): 22/31 (71%) compared with 9/35 (26%) before training. The teams that did not receive specific teamwork training (TW-) used less directed commands after training: 13/20 (65%) compared with 31/32 (97%) before training. | 1b |
| **98.**  **Siassakos et al, 2010**  **United Kingdom** | | BJOG: An International Journal of Obstetrics and Gynaecology 117(10):1262-9 | To determine the active ingredients of effective teams, regardless of their training status | All teams received emergency obstetric clinical care training | 114 maternity care professionals randomly allocated to one of four teams from each unit, to make a total of 24 simulation teams | **Secondary analysis** of data from a RCT of training for obstetric emergencies  (Simulation and Fire-drill Evaluation [SaFE])  **Level 3** | There was a strong, highly significant correlation between clinical efficiency score (magnesium administration) and the Generic Teamwork Scores for all three dimensions; team skills score (taub=0.54, p<0.001), team  behavior score (taub=0.41, p=0.001), and the overall teamwork score (taub=0.51, p<0.001). There was a significant negative correlation between the time taken to perform all of the three key clinical actions and teamwork skills scores (recovery position: taub=−0.29, p=0.012; oxygen administration: taub=−0.39, p<0.001; blood sampling: taub=−0.35, p=0.002). There were similar correlations with the overall teamwork score (recovery position: taub=−0.25, p=0.026; O2 administration: taub=−0.41, p<0.001; blood sampling: taub=−0.35, p=0.002). For the teamwork behavior scores the correlation was statistically significant for oxygen administration (taub=−0.28, p=0.014) and blood sampling (taub=−0.35, p=0.002) but not for recovery position. | 1b |
| **99.**  **Siassakos et al, 2011**  **United Kingdom** | | BJOG: An International Journal of Obstetrics and Gynaecology 118(5):596-607 | To determine whether team performance in a simulated emergency is related to generic teamwork skills and behavior | Eclampsia, shoulder dystocia and postpartum hemorrhage management was assessed only | Six secondary and tertiary maternity units in Southwest England. Participants: 140 healthcare professionals, in 24 teams | **Secondary analysis**  Data from a RCT of training for obstetric emergencies  (Simulation and Fire-drill Evaluation [SaFE])  **Level 3** | The clinical conduct of a simulated emergency was strongly linked to generic measures of teamwork. There was significant positive correlation between clinical efficiency and teamwork scores across all three dimensions; skills (taub=0.54, p< .001), behavior (taub=0.41, p=0.001), and overall score (taub=0.51, p<0.001). Better teams administered the essential drug 2.5 minutes more quickly (p<0.001). | 1b |
| **100.**  **Deering et al, 2004**  **USA** | | Obstetrics and Gynecology 103(6):1224-8 | To determine whether a simulation training scenario improves resident competency in the management of shoulder dystocia. | Management of shoulder dystocia. | 33 residents (16 trained, 17 untrained) | **Randomized controlled trial**  Residents from 2 training programs participated in this study.  The residents were block-randomized by year-group to a training session on shoulder dystocia management that used an obstetric birthing simulator or to a control group with no specific training.  Trained residents and control subjects were subsequently tested on a standardized shoulder dystocia scenario, that were digitally recorded.  An independent external assessor graded and rated the resident's performance with a standardized evaluation sheet.  Statistical analysis included the Student *t* test, [chi]^2^, and regression analysis, as appropriate.  **Level 3** | 1). Trained residents had significantly higher scores in all evaluation categories, including timelines of their interventions, performance of maneuvers, and overall performance.  2). They also performed the delivery in a shorter time than control subjects (61 versus 146 seconds, P .003). | 1 |
| **101.**  **Van de Ven J et al, 2016**  **Netherlands** | | The Journal of Maternal-Fetal and Neonatal Medicine 29(19):3167-71 | To evaluate the effectiveness of simulation team training for the management of shoulder dystocia in a teaching hospital | Simulation team shoulder dystocia training | Not specified. | **Retrospective cohort study**, in a 38-month period before-after implementation of team training.  **Primary outcomes:**  1). number of reported cases of shoulder dystocia  2). as well as fetal injury that occurred from it.  **Secondary outcome:**  Documentation of maneuvers used to alleviate shoulder dystocia.  **Levels 3, 4** | 3492 term vaginal cephalic deliveries were compared with 3496 deliveries before and after team training. Incidence of shoulder dystocia increased from 51 to 90 cases (RR 1.8 (95% CI: 1.3–2.5)).  Fetal injury occurred in 16 and eight cases, respectively (RR 0.50 (95% CI: 0.21–1.2)).  Before team training started, the all-fours maneuver was never used, while after team training it was used in 41 of 90 cases (45%).  Proper documentation of all maneuvers used to alleviate shoulder dystocia significantly increased after team training (RR 1.6 (95% CI: 1.05–2.5)).  Simulation team training increased the frequency of shoulder dystocia, facilitated implementation of the all-fours technique, improved documentation of delivery notes and may have a beneficial effect on the number of children injured due to shoulder dystocia. |  |
| **102.**  **York et al, 2014**  **USA** | | Medical Teacher 36(11):958-64 | To evaluate caesarean surgical training using computer-enhanced visual learning | An online module presented caesarean deliveries as a series of components using text, audio, video and animation | 12 first year obstetric residents | Online self-directed training module, intra operative assessment and feedback over 1 year.  Procedures up to each resident’s 70th case were analyzed by grouping cases in 10s (cases 1-10 and 11-20) or deciles  **Levels 2, 3** | 406 unique observed caesarean deliveries were performed.  Surgical skill acquisition plateaued by cases 21-30, Procedural performance, independent of resident, also improved significantly by decile (x2 (6) =186.95, <0.001), plateauing by decile 4 (cases 31-40) Through the period operative time decreased by 3.84 minutes (p=0.006) | 2b |
